# Supplementary material for: Inequalities in the uptake of, adherence to and effectiveness of behavioural weight management interventions: systematic review protocol
Source: BMJ Open. 2020 Nov 14;10(11):e039518. doi: 10.1136/bmjopen-2020-039518 (PMC7668382; doi:10.1136/bmjopen-2020-039518)
Supplement: Supplementary data [file bmjopen-2020-039518supp002.pdf]

Search Strategies

Cochrane Central Register of Controlled Trials (CENTRAL)
#1 (weight or adipos*):ti or (obesity or obese or overweight or "weight loss"):ti,ab,kw
#2 behavio*:ti,ab,kw
#3 counsel*.ti,ab,kw
#4 cognitive:ti,ab,kw
#5 (diet* or nutrition*):ti,ab,kw
#6 (weightwatcher* or (weight next watcher*)):ti,ab,kw
#7 "physical activity":ti,ab,kw
#8 exercise:ti,ab,kw
#9 (lifestyle or "life style"):ti,ab,kw next (modification* or intervention*):ti,ab,kw
#10 (or #2-#9)
#11 #1 and #10
#12 "weight loss":ti,ab,kw next (intervention* or program* or trial*):ti,ab,kw
#13 (weight next reduc*):ti,ab,kw next (intervention* or program* or trial*):ti,ab,kw
#14 "weight management":ti,ab,kw next (intervention* or program* or trial*):ti,ab,kw
#15 "weight control":ti,ab,kw next (intervention* or program* or trial*):ti,ab,kw
#16 ("weight loss maintenance" next (intervention* or program* or trial*)):ti,ab,kw
#17 (or #11-#16)
#18 (child* or adolescen* or pediatric* or paediatric*)
#19 adult*
#20 (#18 not #19)
#21 (#17 not #20) Publication Year from 2017 to 2020, in Trials

Ovid Medline [ALL KQ]
1 Obesity/
2 Obesity, Morbid/
3 Overweight/
4 Obesity, Metabolically Benign/
5 Weight loss/
6 obes$.ti.
7 overweight.ti.
8 weight.ti.
9 (adipos$ or body fat).ti.
10 (obes$ or overweight or weight loss).ti,ab.
11 limit 10 to ("in data review" or in process or "pubmed not medline")
12 1 or 2 or 3 or 4 or 5 or 6 or 7 or 8 or 9 or 11
13 Weight Reduction Programs/
14 Behavior Therapy/
15 Cognitive Therapy/
16 Counseling/
17 Directive Counseling/
18 Self-Help Groups/
19 counsel$.ti,ab.
20 (behav$ adj3 (therap$ or program$ or intervention$)).ti,ab.
21 Health Education/
22 Diet, Reducing/
23 Diet, Fat-Restricted/
24 Caloric Restriction/
25 Diet Therapy/
26 (diet$ adj counsel$).ti,ab.
27 (diet$ adj education$).ti,ab.
28 (nutrition$ adj counsel$).ti,ab.
29 (nutrition$ adj education$).ti,ab.
30 (nutrition$ adj intervention$).ti,ab.
31 (diet$ adj (modif$ or therapy or intervention$ or strateg$)).ti,ab.
32 ((diet or dieting or slim$) adj (club$ or organi?ation$)).ti,ab.
33 (weight reduc$ adj diet$).ti,ab.
34 (weightwatcher$ or weight watcher$).ti,ab.
35 Exercise/
36 Exercise Therapy/
37 Motor Activity/
38 Physical Conditioning, Human/
39 Physical Fitness/
40 physical activity.ti,ab.
41 (exercise adj3 (therap$ or program$ or intervention$)).ti,ab.
42 ((lifestyle or life style) adj (modification$ or intervention$)).ti,ab.
43 13 or 14 or 15 or 16 or 17 or 18 or 19 or 20 or 21 or 22 or 23 or 24 or 25 or 26 or 27 or 28 or 29 or 30 or 31 or 32 or 33 or 34 or 35 or 36 or 37 or 38 or 39 or 40 or 41 or 42
44 12 and 43
45 Obesity/dh, th, dt, rh [Diet Therapy, Therapy, Drug Therapy, Rehabilitation]
46 Obesity, Morbid/dh, th, dt, rh
47 Overweight/dh, th, dt, rh
48 (weight loss adj (intervention$ or program$ or trial$)).ti,ab.
49 (weight reduc$ adj (intervention$ or program$ or trial$)).ti,ab.
50 (weight management adj (intervention$ or program$ or trial$)).ti,ab.

51 (weight control adj (intervention$ or program$ or trial$)).ti,ab.
52 (weight loss maintenance adj (intervention$ or program$ or trial$)).ti,ab.
53 44 or 45 or 46 or 47 or 48 or 49 or 50 or 51 or 52
54 limit 53 to "all child (0 to 18 years)"
55 limit 53 to "all adult (19 plus years)"
56 54 not 55
57 53 not 56
58 limit 57 to animals
59 limit 57 to humans
60 58 not 59
61 57 not 60
62 clinical trials as topic/ or controlled clinical trials as topic/ or randomized controlled trials as topic/ or meta-analysis as topic/
63 (clinical trial or controlled clinical trial or meta analysis or randomized controlled trial).pt.
64 Random$.ti,ab.
65 control groups/ or double-blind method/ or single-blind method/
66 clinical trial$.ti,ab.
67 controlled trial$.ti,ab.
68 meta analy$.ti,ab.
69 62 or 63 or 64 or 65 or 66 or 67 or 68
70 61 and 69
71 limit 70 to english language
72 limit 71 to yr="2017 -Current"

PsycInfo
1 obesity
2 obese
3 overweight
4 weight loss
5 1 or 2 or 3 or 4
6 weight control/
7 behavior therapy/
8 cognitive behavior therapy/
9 cognitive therapy/
10 Cognitive Techniques/
11 Behavior Modification/
12 Behavior Change/
13 Motivational Interviewing/
14 counseling/
15 counselling.id.
16 Diets/
17 Dietary Restraint/
18 Exercise/
19 Physical Activity/
20 Aerobic Exercise/
21 Walking/
22 6 or 7 or 8 or 9 or 10 or 11 or 12 or 13 or 14 or 15 or 16 or 17 or 18 or 19 or 20 or 21
23 5 and 22
24 random$.ti,ab,id,hw.
25 placebo$.ti,ab,hw,id.
26 controlled trial$.ti,ab,id,hw.
27 clinical trial$.ti,ab,id,hw.
28 meta analy$.ti,ab,hw,id.
29 metaanaly$.ti,ab,hw,id.
30 24 or 25 or 26 or 27 or 28 or 29
31 23 and 30
32 limit 31 to ("300 adulthood <age 18 yrs and older>" or 320 young adulthood <age 18 to 29 yrs> or 340 thirties <age 30 to 39 yrs> or 360 middle age <age 40 to 64 yrs> or "380 aged <age 65 yrs and older>" or "390 very old <age 85 yrs and older>")
33 limit 68 to (english language and yr="2010 -Current")
