# Supplementary material for: Inequalities in the uptake of, adherence to and effectiveness of behavioural weight management interventions: systematic review protocol
Source: BMJ Open. 2020 Nov 14;10(11):e039518. doi: 10.1136/bmjopen-2020-039518 (PMC7668382; doi:10.1136/bmjopen-2020-039518)
Supplement: Author's manuscript [file bmjopen-2020-039518.draft_revisions.pdf]

BMJ Open is committed to open peer review. As part of this commitment we make the peer review history of every article we publish publicly available.

When an article is published we post the peer reviewers' comments and the authors' responses online. We also post the versions of the paper that were used during peer review. These are the versions that the peer review comments apply to.

The versions of the paper that follow are the versions that were submitted during the peer review process. They are not the versions of record or the final published versions. They should not be cited or distributed as the published version of this manuscript.

BMJ Open is an open access journal and the full, final, typeset and author-corrected version of record of the manuscript is available on our site with no access controls, subscription charges or pay-per-view fees (http://bmjopen.bmj.com).

If you have any questions on BMJ Open's open peer review process please email info.bmjopen@bmj.com

BMJ Open

Inequalities in the uptake of, adherence to and effectiveness of behavioural weight management interventions: systematic review protocol

Journal:	*BMJ Open*
Manuscript ID	bmjopen-2020-039518
Article Type:	Protocol
Date Submitted by the Author:	17-Apr-2020
Complete List of Authors:	Birch, Jack; University of Cambridge School of Clinical Medicine, MRC Epidemiology Unit<br>Griffin, Simon; University of Cambridge School of Clinical Medicine, MRC Epidemiology Unit; University of Cambridge School of Clinical Medicine, Primary Care Unit, Institute of Public Health<br>Kelly, Mike; University of Cambridge School of Clinical Medicine, Primary Care Unit, Institute of Public Health<br>Ahern, Amy L.; University of Cambridge Department of Public Health and Primary Care, MRC Epidemiology
Keywords:	PRIMARY CARE, PUBLIC HEALTH, PREVENTIVE MEDICINE

SCHOLARONE™
Manuscripts

I, the Submitting Author has the right to grant and does grant on behalf of all authors of the Work (as defined in the below author licence), an exclusive licence and/or a non-exclusive licence for contributions from authors who are: i) UK Crown employees; ii) where BMJ has agreed a CC-BY licence shall apply, and/or iii) in accordance with the terms applicable for US Federal Government officers or employees acting as part of their official duties; on a worldwide, perpetual, irrevocable, royalty-free basis to BMJ Publishing Group Ltd ("BMJ") its licensees and where the relevant Journal is co-owned by BMJ to the co-owners of the Journal, to publish the Work in this journal and any other BMJ products and to exploit all rights, as set out in our licence.

The Submitting Author accepts and understands that any supply made under these terms is made by BMJ to the Submitting Author unless you are acting as an employee on behalf of your employer or a postgraduate student of an affiliated institution which is paying any applicable article publishing charge ("APC") for Open Access articles. Where the Submitting Author wishes to make the Work available on an Open Access basis (and intends to pay the relevant APC), the terms of reuse of such Open Access shall be governed by a Creative Commons licence – details of these licences and which Creative Commons licence will apply to this Work are set out in our licence referred to above.

Other than as permitted in any relevant BMJ Author's Self Archiving Policies, I confirm this Work has not been accepted for publication elsewhere, is not being considered for publication elsewhere and does not duplicate material already published. I confirm all authors consent to publication of this Work and authorise the granting of this licence.

For peer review only

Inequalities in the uptake of, adherence to and effectiveness of behavioural weight management interventions: systematic review protocol

Jack M. Birch[1], Simon J. Griffin[1, 2], Michael P Kelly[2], Amy L. Ahern[1]

Corresponding author

Jack M. Birch

MRC Epidemiology Unit, University of Cambridge School of Clinical Medicine, Box 285 Institute of Metabolic Science, Cambridge Biomedical Campus, CB2 0QQ

jack.birch@mrc-epid.cam.ac.uk

Author affiliations

[1] MRC Epidemiology Unit, University of Cambridge, Cambridge, UK.

[2] Primary Care Unit, Institute of Public Health, University of Cambridge, Cambridge, UK.

Full text word count 3767/4000 words

For peer review only - http://bmjopen.bmj.com/site/about/guidelines.xhtml

ABSTRACT

Introduction: It has been suggested that interventions focusing on individual behaviour change, such as behavioural weight management interventions, may exacerbate health inequalities. These intervention-generated inequalities may occur at different stages, including intervention uptake, adherence and effectiveness. We will synthesise evidence on how different measures of inequality moderate the uptake, adherence and effectiveness of behavioural weight management interventions in adults.

Methods and analysis: We will update a previous systematic literature review from the US Preventive Services Taskforce to identify trials of behavioural weight management interventions in adults aged 18 and over that were, or could feasibly be, conducted in or recruited from primary care. Medline, Cochrane database (CENTRAL) and PsycINFO will be searched. Only RCTs and cluster-RCTs will be included. Two investigators will independently screen articles for eligibility and conduct risk of bias assessment. We will curate publication families for eligible trials. The PROGRESS-Plus acronym (place of residence, race/ethnicity, occupation, gender, religion, education, socioeconomic status, social capital, plus other discriminating factors) will be used to consider a comprehensive range of health inequalities. Data on trial uptake, intervention adherence, weight change, and PROGRESS-Plus related-data will be extracted. Data will be synthesised narratively. We will present a Harvest plot for each PROGRESS-Plus criteria and whether each trial found a negative, positive or no health inequality gradient. We will also identify potential sources of unpublished original research data on these factors which can be synthesised through a future individual participant data (IPD) meta-analysis.

Ethics and dissemination: Ethical approval is not required as no primary data are being collected. The completed systematic review will be disseminated in a peer-reviewed journal, at conferences, and contribute to the lead author's PhD thesis. Authors of trials included in the completed systematic review may be invited to collaborate on a future IPD meta-analysis.

PROSPERO registration number: Currently under review.

For peer review only

For peer review only - http://bmjopen.bmj.com/site/about/guidelines.xhtml

ARTICLE SUMMARY

Strengths and limitations of the review

- A comprehensive search strategy, which has previously been validated in the literature, will be used to identify relevant trials.
- Only RCTs and cluster RCTs will be included
- There is likely heterogeneity in measures of PROGRESS-Plus criteria used, as well as limited publication of data, which is likely to prevent a meta-analysis being conducted.
- A description of existing data that relate to inequalities in behavioural weight management trials, and where they occur will be provided, enabling future meta-analysis of individual participant data.
- Where data permit, subgroup analysis of association or interaction between the PROGRESS-Plus criteria and trial uptake, adherence and effectiveness will be presented in a Harvest plot.

For peer review only

For peer review only - http://bmjopen.bmj.com/site/about/guidelines.xhtml

INTRODUCTION

Rationale

Overweight and obesity are associated with an increased risk of a number of non-communicable diseases such as type 2 diabetes, cardiovascular disease and some cancers (including post-menopausal breast, bowel and oesophageal).[1, 2] People living with overweight and obesity have greater all-cause mortality compared to those within a healthy weight range.[3] There are known health inequalities by place of residence, ethnicity, occupation, sex, religion, education, socioeconomic status [SES], social capital and other factors such as disability and sexual orientation (PROGRESS-Plus).[4] Observational research suggests that inequalities in overweight and obesity exist across several of these criteria, such as SES and education,[5-10] although these measures are generally more predictive of obesity in women than men.[9, 11]

Both 'upstream' and 'downstream' interventions are needed to reduce the prevalence of obesity through primary prevention and treatment for those living with overweight and obesity. There is suggestion that 'upstream' interventions, i.e. those aimed at a population-level and requiring little personal agency, are the most equitable,[12] and may reduce inequalities in overweight and obesity prevalence. On the other hand, 'downstream' interventions, targeted at high-risk groups and individuals (such as those who already have overweight or obesity) that require high personal agency are likely to be inequitable. Inequitable interventions may exacerbate health inequalities, if they are less effective at reducing overweight and obesity prevalence in disadvantaged groups. Behavioural weight management [BWM] interventions, such as those provided in or referred to from primary care, require a high level of personal agency as participants are required to attend and be engaged with an intervention for it to be effective.[13] Hence, BWM interventions may inadvertently exacerbate health inequalities.

The overall effectiveness of BWM interventions was considered in a systematic review and meta-analysis for the United States Preventive Services Task Force [USPSTF].[14] The review considered behavioural weight loss [BWL] and behavioural weight loss maintenance [BWLM] interventions, as well as pharmacological weight loss and weight loss maintenance interventions. It found that primary care-relevant BWL interventions were associated with greater mean weight loss at 12-18 months when compared to a control, whilst BWLM interventions are effective at preventing weight regain. Moderation of effectiveness by any of the PROGRESS-Plus criteria was not considered, although narrative comment was made about the reporting of ethnicity and SES. Unless a specific ethnicity was targeted in the intervention, the authors found that ethnicity and SES were not well reported. Where ethnicity and SES were reported, the majority of participants were white and of mid-to-high SES. Income, employment and/or occupation were the most frequently reported measures of SES.

We identified one previous systematic review from Hillier-Brown *et al* that considered the effectiveness of individual, community and societal-level interventions at reducing socioeconomic inequalities in obesity.[11] The individual (n=5) and community interventions (n=12) included in the systematic review were similar to the behavioural interventions included in the USPSTF review. They defined individual-level interventions as being conducted in a healthcare, research or home setting and delivered one-to-one. Community-level interventions were defined as being delivered to a group and taking place in community settings such as in community or sports centres. The review found that, for individual-level interventions, evidence for reducing inequalities in obesity among adults was only found in tailored weight loss programmes targeted at low-income groups, particularly those in primary care settings, rather than for 'universal' interventions. Evidence was generally only for short term outcomes (up to 9 months). Community-level interventions showed positive effects up to 3 months, although there

For peer review only - http://bmjopen.bmj.com/site/about/guidelines.xhtml

was no evidence for longer term positive effects. Meanwhile, there was little evidence for the impact of societal-level ('upstream') interventions on inequalities in obesity among adults, and the included evidence was of low quality.

There are some limitations of the Hillier-Brown *et al* review. A meta-analysis was not conducted, due to heterogeneity of the studies. Whilst a highly sensitive search strategy was used, only literature that reported differential effects by a measure of SES were included. This meant that interventions which may have collected data on SES but had not included it in analyses reported in a published paper would have been excluded. The authors highlighted that this may have explain why mostly interventions that took a targeted approach to reducing SES inequalities were included; only a minority of studies examined intervention effects across the SES gradient. They also say that this targeted approach "has limitations as even when interventions are effective among low-income groups they are only able to reduce the health inequalities gap, they have little effect on the wider social gradient". Literature published since Hillier-Brown *et al* has considered the effect of universal interventions on health inequalities amongst adults.[15, 16]

The Hillier-Brown *et al* review only considered inequalities in intervention effectiveness. Intervention-generated inequalities may occur at several stages.[12] Firstly, in intervention uptake.[17] This may occur because of differing levels of weight loss service provision by geography or because some groups are less likely to take up the offer of an intervention. Research using the Clinical Practice Research Datalink has found that certain groups, such as those in deprivation, may be more likely to access weight management interventions,[18] suggesting that such interventions may have a positive effect on health inequalities. Secondly, inequalities may occur in the adherence to an intervention.[19] Adherence to an intervention may be affected by certain barriers such as access to transport,[20] insufficient time or other social circumstances. Thirdly, there may be inequalities in outcome –those of a certain socioeconomic position or ethnicity may have similar uptake and adherence to an intervention, but there may be other factors that mean that the intervention is less effective for them than for other people. This may be because the intervention is not appropriately culturally or contextually tailored.

In the current systematic review, we will synthesise literature on inequalities across the uptake, adherence and effectiveness of BWM interventions The lack of reporting or analysis by measures associated with the PROGRESS-Plus criteria identified in both the USPSTF and Hillier Brown *et al's* systematic reviews suggest that it is not possible to fully explore inequalities in the effectiveness using aggregated data from published literature alone. This lack of reporting may have occurred because individual trials may not be sufficiently powered to detect an interaction between moderators such as SES and the outcome; they are likely just to be sufficiently powered to detect the main, overall effect. This systematic review will also identify trials with unpublished data on measures of inequality across the PROGRESS-Plus criteria in order to conduct a future individual participant data [IPD] meta-analysis.

Objectives

The overall aim is to identify and describe inequalities in the update, adherence and effectiveness of BWM interventions. We will meet this aim through the following objectives: 1) to synthesise published literature on how inequalities across different PROGRESS-Plus criteria moderate the uptake, adherence and effectiveness of BWM interventions and; 2) to identify published trials that have unpublished data on how inequalities across different PROGRESS-Plus criteria moderate the uptake, adherence and effectiveness of BWM interventions.

For peer review only - http://bmjopen.bmj.com/site/about/guidelines.xhtml

METHODS

This protocol was written in accordance with the PRISMA-P reporting guidelines for systematic review protocols (supplementary file A).[21]

Study Design

We will conduct a systematic review of published randomised controlled trials [RCTs] of BWM interventions (which includes interventions for both BWL and BWLM). PROGRESS-Plus criteria-related measures and data (outlined in Table 1) will be extracted and evidence regarding their impact on uptake, adherence and effectiveness will be synthesised. Furthermore, we will identify where data relating to the PROGRESS-Plus criteria have been collected and their relationship with uptake, adherence and effectiveness not analysed, to facilitate future IPD meta-analysis.

Initially, relevant literature concerning BWM trials will be extracted from the 2018 USPSTF systematic review of interventions to prevent obesity-related morbidity and mortality in adults.[14] Then, a search of the same databases used in the USPSTF review will be conducted to identify trials published since the search was completed for the USPSTF systematic review on 6th June 2017. We will use the same search strategies and terms as in the original report, but with pharmacological interventions excluded and terms relating to adverse events removed.

For peer review only

Table 1 Definition of PROGRESS-Plus factors

PROGRESS-Plus Factor	Description	Example measures
**Place of residence**	Places, and perceptions of, where individuals live	• Post code<br>• Country, state, region, town or community<br>• Urban/rural<br>• Housing characteristics<br>• Distance to attend weight loss session<br>• Local food environment<br>• 'Walkability'
**Race/ethnicity**	Racial or ethnic group, or other classification of culture, language or nationality status	• Ethnicity classifications<br>• Country of origin<br>• Language<br>• Other classifications of culture
**Occupation**	Occupational situation, patterns of work or features of working environment	• Professional/skilled/unskilled/unemployed<br>• Unemployed/employed/retired<br>• Full time/part time<br>• Manual/non-manual
**Gender/sex**	Gender is self-identified by individuals, incorporating ideas around socially constructed roles and behaviours<br><br>Sex refers to biological and physiological characteristics that define an individual as a man or woman	• Gender<br>• Sex (e.g. male/female classifications)
**Religion**	Religious affiliation or system of religious/spiritual beliefs or values	• Religious denomination
**Education**	Extent and type of education or other formal training	• Years in education<br>• Level of education attained (e.g. for UK: GCSE's, A-Levels, Undergraduate)<br>• Institutions attended (e.g. for USA: high school/some college/college graduate/university)
**Socioeconomic status**	An individual's position within a hierarchical social structure. Measures of socioeconomic status aim to capture access to resources, privilege, power or control	• Indices of Multiple Deprivation (IMD, UK only, Scottish Index of Multiple Deprivation)<br>• Social class<br>• Individual income<br>• Household income<br>• Receiving state welfare (e.g. benefits/free prescriptions in the UK, Medicaid in the USA)

For peer review only - http://bmjopen.bmj.com/site/about/guidelines.xhtml

		• Asset-based measures (e.g. home or car ownership)<br>• Occupation (e.g. occupation class)
**Social capital**	Social capital aims to capture the obligations and benefits conferred upon an individual by their society and social relationships. Can be viewed as a measure of interconnectedness between an individual and their social surroundings or group	• Marital/relationship status (e.g. single, cohabiting)<br>• Household size<br>• Social support<br>• Social networks<br>• Civic participation/group membership<br>• Ability to use technology
**Plus**	Any other factors over an individual's life course that could lead to discrimination. Examples include age, disability and sexual orientation	• Self-reported age in years<br>• Measures of health status and/or quality of life (e.g. EuroQoL, SF-36, EQ5D)<br>• Tests of physical function<br>• Physical or emotional/mental disability<br>• Self-reported sexual orientation (e.g. heterosexual, homosexual, bisexual)

Eligibility criteria

We will select studies according to the criteria outlined below.

Study designs

We will include research articles reporting randomised controlled trials (RCTs) and cluster-RCTs. To mirror the USPSTF review, only studies published in the English language will be included.

Participants

We will include studies of adults aged 18 years and over with overweight or obesity (BMI $\geq$ 25 kg/m^2) who are suitable for BWL or BWLM interventions. Participants may have additional risk factors such as hypertension, dyslipidaemia, impaired glucose tolerance or impaired fasting glucose.

Studies will be excluded if the population: was not selected based on a weight-related measure; had secondary causes of obesity (such as steroid use); selected on the basis of having a chronic disease for which BWL or BWLM is part of disease management; was of pregnant women; was of adults in institutions; or if the intervention was targeted at parents in order to change the behaviour of children.

Interventions

Studies will be included if they were conducted in or recruited from primary care or a healthcare system, or could feasibly be implemented in or referred to from primary care. In the case of the latter, the interventions must be conducted as part of a healthcare setting or be available in the community at a national level, such as commercial weight loss interventions. We will include behavioural interventions that are focused on weight loss or weight loss maintenance. Interventions may be delivered either alone or as part of a multicomponent intervention on wider diet and nutrition, physical activity, sedentary behaviour or a combination of these. The intervention may include (but not limited to): assessment with

For peer review only - http://bmjopen.bmj.com/site/about/guidelines.xhtml

feedback, advice, collaborative goal-setting, assistance, exercise prescriptions (referral to exercise facility or programme), arranging further contacts or provider training.

The delivery of the intervention may be: face-to-face contact, telephone, print materials, or be computer or mobile-phone based technology (such as websites, apps or text messages). There is no restriction on who delivers the intervention.

Interventions of alternative and complementary treatments (e.g. mindfulness) will be excluded. All pharmacological and surgical interventions will be excluded, including in combination with behavioural interventions, unless the trial includes behavioural only and control arms.

Comparators

We will only include trials with a control group. The control group may receive no intervention (wait-list control or usual care) or minimal intervention (such as generic print or electronic materials).

Outcomes and prioritisation

Outcomes will occur at the three stages: uptake, attendance/adherence, and effectiveness.

Differential uptake will be considered at two stages. Firstly, trial uptake will be calculated for each study using the formula:

$$\frac{Participants\ accepting\ invitation\ to\ trial}{Participants\ invited\ to\ trial}$$

Secondly, uptake of the intervention arm. We are considering uptake of the intervention to be ‘attending’ at least one intervention session, the language of which is geared towards to those attending community group interventions such as WW (formerly Weight Watchers). ‘Attendance’ to an online-based intervention would be defined as logging into the online platform at least once. Hence, uptake of the intervention is defined as:

$$\frac{Participants\ attending\ at\ least\ one\ intervention\ session}{Participants\ in\ trial\ arm}$$

The second outcome stage is adherence to the intervention. We will consider adherence for each participant as either a binary variable (adhered versus not adhered) or using the below formula, depending on how adherence is defined in the included studies.

$$\frac{Number\ of\ sessions\ attended}{Number\ of\ sessions\ prescribed}$$

The final outcome stage is effectiveness. This will be assessed at the 12-month follow up using three measures: weight change in kilograms, weight loss of five percent or greater, and change in waist circumference.

Timing (e.g. minimum follow up)

As per the USPSTF review, we will only include studies that measure intervention effectiveness at 12 or 18 months. This is despite the different timing required for each of the outcomes. The uptake outcomes require data at two pre-intervention stages – invitation to trial and baseline – as well as data on percentage of participants attending at least one session of an intervention. The adherence outcome

For peer review only - http://bmjopen.bmj.com/site/about/guidelines.xhtml

will require data from baseline until the end of the intervention. Finally, intervention effectiveness will be assessed at 12 months or later from baseline. We will contact authors where data relating to the uptake, adherence and effectiveness outcomes have not been published.

Moderator variables

The moderator variables under consideration are the PROGRESS-Plus criteria. Possible measures of each of these criteria are shown in Table 1, which has been adapted from a systematic review that explored equity in primary care-based physical activity interventions using PROGRESS-Plus [22].

Setting

Eligible studies will have been conducted in primary care, referred from primary care, or be applicable to primary care settings. As per the search performed in the USPSTF review, only studies conducted in countries that were members of the Organisation for Economic Co-operation and Development (as of 2017) are eligible for inclusion. These countries are: Australia, Austria, Belgium, Canada, Chile, Czech Republic, Denmark, Estonia, Finland, France, Germany, Greece, Hungary, Iceland, Israel, Italy, Japan, South Korea, Luxembourg, Mexico, the Netherlands, New Zealand, Norway, Poland, Portugal, Slovakia, Slovenia, Spain, Sweden, Switzerland, Turkey, United Kingdom and the United States.

Information sources and search strategy

Electronic searches

It is anticipated that much of the PROGRESS-Plus data to be extracted will not be reported in the main write up of each BWM intervention RCT. Hence, it is necessary to extract data from all publications associated with each individual RCT. To complete this, we will adopt a similar approach to literature searching as demonstrated by Orkin *et al*.[23]

The search strategy will be completed in two phases. Phase 1 is identifying 'parent' RCTS. These studies will be identified in two ways. Initially, the BWL and BWLM interventions included in the USPSTF report will be extracted. Then, we will conduct an update of the literature search used in the USPSTF report to capture recent published trials using the same databases (Medline, CENTRAL and PsychInfo). Databases will be searched from June 2017 (the last date of the USPSTF report) to February 2020. The search strategy includes the following concepts: 1) overweight and obesity AND 2) BWL/BWLM interventions. The Medline, CENTRAL and PsychInfo search strategies are outlined in supplementary file B. In addition to the databases, reference lists of included published primary research and relevant systematic reviews and meta-analyses will be searched for possible further studies for inclusion.

Phase 2 is to "curate publication families".[23] This involves identifying publications of any type that relate to the parent RCT through electronic database searching. Authors and study identifiers (such as trial name) will be extracted from each parent RCT, which will then be searched for in the same electronic bibliographic databases as phase 1. Each publication family will be considered as one study.

Study records

Data management and study selection

Search results will be imported into Endnote X7 bibliographic software, where duplicates will be removed. The literature will then be loaded onto Covidence systematic review software (Veritas Health

For peer review only - http://bmjopen.bmj.com/site/about/guidelines.xhtml

Innovation, Melbourne, Australia), and title and abstract screening conducted. Piloting of 500 articles will be conducted with minimum two investigators, where differences in interpretation of the inclusion criteria will be discussed between the investigators in order to achieve consistency in the review process. Once this has been completed, the remaining titles and abstracts will be screened for inclusion by minimum two investigators independently. Full-text articles identified as being potentially relevant to the research questions will be accessed and screened by minimum two investigators. Any conflicts will be discussed and resolved by a third reviewer if agreement cannot be reached. For articles excluded at full-text stage, reasons for exclusion will be recorded.

Multiple articles reporting the same study will all be included and amalgamated to ensure all the best available data are used. A PRISMA flow chart will be reported to visualise the study selection.[24]

Data items

For studies highlighted as eligible for inclusion from the USPSTF report, and those that fulfil the inclusion criteria from our subsequent searches, we will extract data from the reports onto a data extraction form. To ensure that an appropriate breadth and depth of detail is captured, the data extraction form will be based on the Cochrane Public Health Group data extraction form,[25] the Consolidated Standards of Reporting Trials 2010 statement,[26] the Template for Intervention Description and Replication checklist and the PROGRESS-Plus criteria.[4, 27]

The following data will be extracted from the studies:

- General information (study authors, publication year, country and source of funding)
- Study information (study aim, design, recruitment location and method, randomisation, blinding and allocation concealment)
- Participant information (measures associated with the PROGRESS-Plus criteria as outlined in Table 1)
- Intervention information (content, delivery method, group or individual-level, duration, setting, profession of person delivering intervention)
- Comparator information (control/usual care, content, delivery method, group or individual-level, duration, setting, profession of person delivering intervention)
- Uptake (number of participants invited to trial, number of participants accepting invite, number of participants randomised to intervention arm, number of participants attending $\geq$ 1 session), including impact of PROGRESS-Plus criteria on uptake
- Attrition/adherence/attendance, including impact of PROGRESS-Plus criteria on these
- Outcomes (outcomes studies, self-report or objective, follow-up duration, statistical analyses, intervention effect sizes), including impact of PROGRESS-Plus criteria on outcomes

Risk of bias in individual studies

We will use Cochrane's risk of bias tool for randomised trials (RoB 2) to assess risk of bias across individual studies.[28] The tool covers six domains of possible bias: the randomisation process; allocation concealment; participant and trial personnel blinding; blinding of outcome assessment; incomplete outcome data; and selective reporting. Each domain is given a ranking of 'low risk', 'high risk', or 'unclear. This will be performed independently by at least two study authors. Where disagreements occur, these will be discussed between authors to reach consensus. A third reviewer will be consulted if agreement cannot be reached. Other possible sources of bias that does not fall within RoB 2's six domains will be noted by reviewers, and commented on if appropriate in the final review. Reviewers will not be blinded to study information (such as study author, institution or journal). Results

For peer review only - http://bmjopen.bmj.com/site/about/guidelines.xhtml

of the risk of bias assessments will be presented in a summary figure outlining a study's overall risk of bias as well as the risk of bias in each domain.

Data synthesis

Narrative synthesis and Harvest plots

We anticipate that there will be insufficient data to conduct a meta-analysis, therefore, the primary methods of data synthesis will be narrative analysis and using Harvest plots.[29] Harvest plots were proposed by Ogilvie *et al* as a method for synthesising evidence of the differential effectiveness of population-level public interventions,[29] but have been used in systematic reviews of various intervention types since.[22, 30-34] Even where there is heterogeneity in measures used, Harvest plots allow for all available and relevant data to be used and presented.[29, 35, 36] Several study features can be graphically demonstrated on a single plot, such as study quality, statistical significance and sample size. We will present a Harvest plot for each PROGRESS-Plus criteria and whether each trial found a negative, positive or no health inequality gradient; sample size of each study group; and whether the trial considered an intervention or interaction effect on the health inequality gradient.

Meta-analysis

Should there be sufficient data to conduct a meta-analysis, then the meta-analysis will consider two questions: *are the PROGRESS-Plus criteria associated with the amount of weight loss achieved following BWM intervention?* and *do the PROGRESS-Plus criteria moderate the effectiveness of BWM interventions?*. Odds or risk ratios would be pooled for each question; the first question assesses if there is an association between the PROGRESS-Plus criteria and weight loss, the second question considers if there is an interaction. The data would be analysed using Stata v16 (StataCorp. 2019. College Station, TX: StataCorp LLC), using a random-effects meta-analysis.

Statistical heterogeneity will be assessed using the I^2 statistic and its 95% confidence interval. The I^2 statistic will be interpreted against the following categorisations: 0% to 40% might not be important; 30% to 60% may represent moderate heterogeneity; 50% to 90% may represent substantial heterogeneity and; 75% to 100% is likely considerable heterogeneity.[37] Publication bias will be considered through the use of a funnel plot.

Patient and public involvement

A patient and public involvement representative reviewed a lay summary of our proposed plan for the systematic review. Feedback was received on the review's aims and definitions of the PROGRESS-Plus criteria. Once the review has been completed, feedback will be sought from the patient and public involvement representatives about the interpretation of findings and plans for an individual participant data meta-analysis.

ETHICS AND DISSEMINATION

Ethical approval is not required as only aggregate data are going to be acquired, and will be used for the purpose for which they were originally collected for. Ethical approval for each trial to be included will have been sought by the original investigators. This systematic review and meta-analysis will follow the PRISMA statement.[24]

Inequalities in overweight and obesity, and in health promotion interventions, are widely recognised. However, inequalities in behavioural weight loss interventions delivered or referred to from primary

For peer review only - http://bmjopen.bmj.com/site/about/guidelines.xhtml

care (or similar) have not yet been considered in a systematic review. This review will identify data on where inequalities in weight loss interventions occur (i.e. in which PROGRESS-Plus criteria), and at what stage (uptake, adherence or effectiveness). We anticipate the completed systematic review will be published in a scientific journal, presented at conferences and contribute to the lead author's PhD thesis. The review findings will contribute towards the consideration of intervention-generated inequalities by researchers, policy makers and healthcare and public health practitioners.

Acknowledgements

The authors would like to thank Hazel Patel, the patient and public involvement representative, who commented on the lay summary of our proposed plan, for their contribution in the development of this research.

Competing interests

ALA is principal investigator on two publicly funded (NIHR, MRC) trials where the intervention is provided by WW (formerly Weight Watchers) at no cost. MPK has undertaken consultancy for Slimming World, and led the clinical and public health guidelines development for NICE from 2005 until 2014.

Author contributions

JMB conceived and designed the study, developed the search strategy and drafted the manuscript. SJG conceived the study, contributed to study design and reviewed drafts of the manuscript. MPK contributed to study design and reviewed drafts of the manuscript. ALA conceived the study, contributed to study design and reviewed drafts of the manuscript. All authors have reviewed the manuscript and approved the final version for publication.

Funding

JMB, SJG and ALA are supported by the Medical Research Council (MRC) (Grant MC_UU_12015/4). The University of Cambridge has received salary support in respect of SJG from the National Health Service in the East of England through the Clinical Academic Reserve.

For peer review only

For peer review only - http://bmjopen.bmj.com/site/about/guidelines.xhtml

REFERENCES

1. Lauby-Secretan B, Scoccianti C, Loomis D, et al. Body Fatness and Cancer — Viewpoint of the IARC Working Group. New England Journal of Medicine. 2016;375(8):794-8.
2. Forouzanfar MH, Afshin A, Alexander LT, et al. Global, regional, and national comparative risk assessment of 79 behavioural, environmental and occupational, and metabolic risks or clusters of risks, 1990–2015: a systematic analysis for the Global Burden of Disease Study 2015. The Lancet. 2016;388(10053):1659-724.
3. Di Angelantonio E, Bhupathiraju SN, Wormser D, et al. Body-mass index and all-cause mortality: individual-participant-data meta-analysis of 239 prospective studies in four continents. The Lancet. 2016;388(10046):776-86.
4. O'Neill J, Tabish H, Welch V, et al. Applying an equity lens to interventions: using PROGRESS ensures consideration of socially stratifying factors to illuminate inequities in health. Journal of clinical epidemiology. 2014;67(1):56-64.
5. Newton S, Braithwaite D, Akinyemiju TF. Socio-economic status over the life course and obesity: Systematic review and meta-analysis. PLoS One. 2017;12(5):e0177151-e.
6. Loring B, Robertson A. Guidance for addressing inequities in overweight and obesity. Copenhagen: Word Health Organization Regional Office for Europe; 2014.
7. Vilhelmsson A, Östergren P-O. Reducing health inequalities with interventions targeting behavioral factors among individuals with low levels of education - A rapid review. PLoS One. 2018;13(4):e0195774-e.
8. Clarke P, O'Malley PM, Johnston LD, et al. Social disparities in BMI trajectories across adulthood by gender, race/ethnicity and lifetime socio-economic position: 1986-2004. Int J Epidemiol. 2009;38(2):499-509.
9. El-Sayed AM, Scarborough P, Galea S. Unevenly distributed: a systematic review of the health literature about socioeconomic inequalities in adult obesity in the United Kingdom. BMC public health. 2012;12(1):18.
10. Rohrmann S, Steinbrecher A, Linseisen J, et al. The association of education with long-term weight change in the EPIC-PANACEA cohort. European Journal of Clinical Nutrition. 2012;66(8):957-63.
11. Hillier-Brown FC, Bambra CL, Cairns JM, et al. A systematic review of the effectiveness of individual, community and societal-level interventions at reducing socio-economic inequalities in obesity among adults. International journal of obesity (2005). 2014;38(12):1483-90.
12. White M, Adams J, Heywood P. How and why do interventions that increase health overall widen inequalities within populations? . In: Babones SJ, editor. Social inequality and public health. Bristol: Policy Press; 2009.
13. Adams J, Mytton O, White M, et al. Why Are Some Population Interventions for Diet and Obesity More Equitable and Effective Than Others? The Role of Individual Agency. PLOS Medicine. 2016;13(4):e1001990.
14. LeBlanc ES, Patnode CD, Webber EM, et al. Behavioral and Pharmacotherapy Weight Loss Interventions to Prevent Obesity-Related Morbidity and Mortality in Adults: Updated Evidence Report and Systematic Review for the US Preventive Services Task Force. Jama. 2018;320(11):1172-91.
15. Graham J, Tudor K, Jebb SA, et al. The equity impact of brief opportunistic interventions to promote weight loss in primary care: secondary analysis of the BWeL randomised trial. BMC Med. 2019;17(1):51-.
16. Greaves C, Gillison F, Stathi A, et al. Waste the waist: a pilot randomised controlled trial of a primary care based intervention to support lifestyle change in people with high cardiovascular risk. International Journal of Behavioral Nutrition and Physical Activity. 2015;12(1):1.
17. Ahern AL, Aveyard P, Boyland EJ, et al. Inequalities in the uptake of weight management interventions in a pragmatic trial: an observational study in primary care. British Journal of General Practice. 2016;66(645):e258-e63.
18. Booth HP, Prevost AT, Gulliford MC. Access to weight reduction interventions for overweight and obese patients in UK primary care: population-based cohort study. BMJ Open. 2015;5(1):e006642.

For peer review only - http://bmjopen.bmj.com/site/about/guidelines.xhtml

19. Kure-Biegel N, Schnohr CW, Hindhede AL, et al. Risk factors for not completing health interventions and the potential impact on health inequalities between educational groups – a mixed method study from Denmark. International Journal for Equity in Health. 2016;15(1):54.
20. Austin JL, Smith JE, Gianini L, et al. Attitudinal familism predicts weight management adherence in Mexican–American women. J Behav Med. 2013;36(3):259-69.
21. Moher D, Shamseer L, Clarke M, et al. Preferred reporting items for systematic review and meta-analysis protocols (PRISMA-P) 2015 statement. Systematic Reviews. 2015;4(1):1.
22. Attwood S, van Sluijs E, Sutton S. Exploring equity in primary-care-based physical activity interventions using PROGRESS-Plus: a systematic review and evidence synthesis. The international journal of behavioral nutrition and physical activity. 2016;13:60.
23. Orkin AM, McArthur A, McDonald A, et al. Defining and measuring health equity effects in research on task shifting interventions in high-income countries: a systematic review protocol. BMJ Open. 2018;8(7):e021172.
24. Moher D, Liberati A, Tetzlaff J, et al. Preferred reporting items for systematic reviews and meta-analyses: the PRISMA statement. BMJ. 2009;339.
25. The Cochrane Public Health Group. Cochrane public health group data extraction and assessment template. 2011.
26. Schulz KF, Altman DG, Moher D, et al. CONSORT 2010 Statement: updated guidelines for reporting parallel group randomised trials. BMC Med. 2010;8:18-.
27. Hoffmann TC, Glasziou PP, Boutron I, et al. Better reporting of interventions: template for intervention description and replication (TIDieR) checklist and guide. BMJ. 2014;348:g1687-g.
28. Sterne JAC, Savović J, Page MJ, et al. RoB 2: a revised tool for assessing risk of bias in randomised trials. BMJ. 2019;366:l4898.
29. Ogilvie D, Fayter D, Petticrew M, et al. The harvest plot: A method for synthesising evidence about the differential effects of interventions. BMC Medical Research Methodology. 2008;8(1):8.
30. Romppanen J, Häggman-Laitila A. Interventions for nurses' well-being at work: a quantitative systematic review. Journal of Advanced Nursing. 2017;73(7):1555-69.
31. Moore GF, Littlecott HJ, Turley R, et al. Socioeconomic gradients in the effects of universal school-based health behaviour interventions: a systematic review of intervention studies. BMC Public Health. 2015;15(1):907.
32. Wijndaele K, Lakshman R, Landsbaugh JR, et al. Determinants of Early Weaning and Use of Unmodified Cow's Milk in Infants: A Systematic Review. Journal of the American Dietetic Association. 2009;109(12):2017-28.
33. Kovacs E, Strobl R, Phillips A, et al. Systematic Review and Meta-analysis of the Effectiveness of Implementation Strategies for Non-communicable Disease Guidelines in Primary Health Care. J Gen Intern Med. 2018;33(7):1142-54.
34. Nanninga S, Lehne G, Ratz T, et al. Impact of Public Smoking Bans on Social Inequalities in Children's Exposure to Tobacco Smoke at Home: An Equity-Focused Systematic Review. Nicotine & Tobacco Research. 2018;21(11):1462-72.
35. Burns J, Polus S, Brereton L, et al. Looking beyond the forest: Using harvest plots, gap analysis, and expert consultations to assess effectiveness, engage stakeholders, and inform policy. Research Synthesis Methods. 2018;9(1):132-40.
36. Crowther M, Avenell A, MacLennan G, et al. A further use for the Harvest plot: a novel method for the presentation of data synthesis. Research synthesis methods. 2011;2(2):79-83.
37. Ryan R. Cochrane Consumers and Communication Review Group. 'Heterogeneity and subgroup analyses in Cochrane Consumers and Communication Group reviews: planning the analysis at protocol stage. Cochrane Consumers and Communication; 2016. Contract No.: 06/02/2020.

For peer review only - http://bmjopen.bmj.com/site/about/guidelines.xhtml

PRISMA-P (Preferred Reporting Items for Systematic review and Meta-Analysis Protocols) 2015 checklist: recommended items to address in a systematic review protocol*

Section and topic	Item No	Checklist item	Location in text
**ADMINISTRATIVE INFORMATION**			
Title:			
Identification	1a	Identify the report as a protocol of a systematic review	Page 1
Update	1b	If the protocol is for an update of a previous systematic review, identify as such	N/A
Registration	2	If registered, provide the name of the registry (such as PROSPERO) and registration number	Page 2 (abstract)
Authors:			
Contact	3a	Provide name, institutional affiliation, e-mail address of all protocol authors; provide physical mailing address of corresponding author	Page 1
Contributions	3b	Describe contributions of protocol authors and identify the guarantor of the review	Page 13
Amendments	4	If the protocol represents an amendment of a previously completed or published protocol, identify as such and list changes; otherwise, state plan for documenting important protocol amendments	N/A
Support:			
Sources	5a	Indicate sources of financial or other support for the review	Page 13
Sponsor	5b	Provide name for the review funder and/or sponsor	Page 13
Role of sponsor or funder	5c	Describe roles of funder(s), sponsor(s), and/or institution(s), if any, in developing the protocol	Page 13
**INTRODUCTION**			
Rationale	6	Describe the rationale for the review in the context of what is already known	Page 4-5
Objectives	7	Provide an explicit statement of the question(s) the review will address with reference to participants, interventions, comparators, and outcomes (PICO)	Page 5
**METHODS**			
Eligibility criteria	8	Specify the study characteristics (such as PICO, study design, setting, time frame) and report characteristics (such as years considered, language, publication status) to be used as criteria for eligibility for the review	Page 5-10
Information sources	9	Describe all intended information sources (such as electronic databases, contact with study authors, trial registers or other grey literature sources) with planned dates of coverage	Page 10
Search strategy	10	Present draft of search strategy to be used for at least one electronic database, including planned limits, such that it could be repeated	Supplementary file B

For peer review only

Study records:			
Data management	11a	Describe the mechanism(s) that will be used to manage records and data throughout the review	Page 10-11
Selection process	11b	State the process that will be used for selecting studies (such as two independent reviewers) through each phase of the review (that is, screening, eligibility and inclusion in meta-analysis)	Page 11
Data collection process	11c	Describe planned method of extracting data from reports (such as piloting forms, done independently, in duplicate), any processes for obtaining and confirming data from investigators	Page 11
Data items	12	List and define all variables for which data will be sought (such as PICO items, funding sources), any pre-planned data assumptions and simplifications	Page 11
Outcomes and prioritization	13	List and define all outcomes for which data will be sought, including prioritization of main and additional outcomes, with rationale	Page 9-10
Risk of bias in individual studies	14	Describe anticipated methods for assessing risk of bias of individual studies, including whether this will be done at the outcome or study level, or both; state how this information will be used in data synthesis	Page 11-12
Data synthesis	15a	Describe criteria under which study data will be quantitatively synthesised	Page 12
	15b	If data are appropriate for quantitative synthesis, describe planned summary measures, methods of handling data and methods of combining data from studies, including any planned exploration of consistency (such as I^2, Kendall's τ)	Page 12
	15c	Describe any proposed additional analyses (such as sensitivity or subgroup analyses, meta-regression)	Page 12
	15d	If quantitative synthesis is not appropriate, describe the type of summary planned	Page 12
Meta-bias(es)	16	Specify any planned assessment of meta-bias(es) (such as publication bias across studies, selective reporting within studies)	Page 12
Confidence in cumulative evidence	17	Describe how the strength of the body of evidence will be assessed (such as GRADE)	

*** It is strongly recommended that this checklist be read in conjunction with the PRISMA-P Explanation and Elaboration (cite when available) for important clarification on the items. Amendments to a review protocol should be tracked and dated. The copyright for PRISMA-P (including checklist) is held by the PRISMA-P Group and is distributed under a Creative Commons Attribution Licence 4.0.**

From: Shamseer L, Moher D, Clarke M, Ghersi D, Liberati A, Petticrew M, Shekelle P, Stewart L, PRISMA-P Group. Preferred reporting items for systematic review and meta-analysis protocols (PRISMA-P) 2015: elaboration and explanation. BMJ. 2015 Jan 2;349(jan02 1):g7647.

For peer review only

Search Strategies

Cochrane Central Register of Controlled Trials (CENTRAL)

#1 (weight or adipos*):ti or (obesity or obese or overweight or "weight loss"):ti,ab,kw
#2 behavio*:ti,ab,kw
#3 counsel*.ti,ab,kw
#4 cognitive:ti,ab,kw
#5 (diet* or nutrition*):ti,ab,kw
#6 (weightwatcher* or (weight next watcher*)):ti,ab,kw
#7 "physical activity":ti,ab,kw
#8 exercise:ti,ab,kw
#9 (lifestyle or "life style"):ti,ab,kw next (modification* or intervention*):ti,ab,kw
#10 (or #2-#9)
#11 #1 and #10
#12 "weight loss":ti,ab,kw next (intervention* or program* or trial*):ti,ab,kw
#13 (weight next reduc*):ti,ab,kw next (intervention* or program* or trial*):ti,ab,kw
#14 "weight management":ti,ab,kw next (intervention* or program* or trial*):ti,ab,kw
#15 "weight control":ti,ab,kw next (intervention* or program* or trial*):ti,ab,kw
#16 ("weight loss maintenance" next (intervention* or program* or trial*)):ti,ab,kw
#17 (or #11-#16)
#18 (child* or adolescen* or pediatric* or paediatric*)
#19 adult*
#20 (#18 not #19)
#21 (#17 not #20) Publication Year from 2017 to 2020, in Trials

For peer review only

Ovid Medline [ALL KQ]
1 Obesity/
2 Obesity, Morbid/
3 Overweight/
4 Obesity, Metabolically Benign/
5 Weight loss/
6 obes$.ti.
7 overweight.ti.
8 weight.ti.
9 (adipos$ or body fat).ti.
10 (obes$ or overweight or weight loss).ti,ab.
11 limit 10 to ("in data review" or in process or "pubmed not medline")
12 1 or 2 or 3 or 4 or 5 or 6 or 7 or 8 or 9 or 11
13 Weight Reduction Programs/
14 Behavior Therapy/
15 Cognitive Therapy/
16 Counseling/
17 Directive Counseling/
18 Self-Help Groups/
19 counsel$.ti,ab.
20 (behav$ adj3 (therap$ or program$ or intervention$)).ti,ab.
21 Health Education/
22 Diet, Reducing/
23 Diet, Fat-Restricted/
24 Caloric Restriction/
25 Diet Therapy/
26 (diet$ adj counsel$).ti,ab.
27 (diet$ adj education$).ti,ab.
28 (nutrition$ adj counsel$).ti,ab.
29 (nutrition$ adj education$).ti,ab.
30 (nutrition$ adj intervention$).ti,ab.
31 (diet$ adj (modif$ or therapy or intervention$ or strateg$)).ti,ab.
32 ((diet or dieting or slim$) adj (club$ or organi?ation$)).ti,ab.
33 (weight reduc$ adj diet$).ti,ab.
34 (weightwatcher$ or weight watcher$).ti,ab.
35 Exercise/
36 Exercise Therapy/
37 Motor Activity/
38 Physical Conditioning, Human/
39 Physical Fitness/
40 physical activity.ti,ab.
41 (exercise adj3 (therap$ or program$ or intervention$)).ti,ab.
42 ((lifestyle or life style) adj (modification$ or intervention$)).ti,ab.
43 13 or 14 or 15 or 16 or 17 or 18 or 19 or 20 or 21 or 22 or 23 or 24 or 25 or 26 or 27 or 28 or 29 or 30 or 31 or 32 or 33 or 34 or 35 or 36 or 37 or 38 or 39 or 40 or 41 or 42
44 12 and 43
45 Obesity/dh, th, dt, rh [Diet Therapy, Therapy, Drug Therapy, Rehabilitation]
46 Obesity, Morbid/dh, th, dt, rh
47 Overweight/dh, th, dt, rh
48 (weight loss adj (intervention$ or program$ or trial$)).ti,ab.
49 (weight reduc$ adj (intervention$ or program$ or trial$)).ti,ab.
50 (weight management adj (intervention$ or program$ or trial$)).ti,ab.

For peer review only

51 (weight control adj (intervention$ or program$ or trial$)).ti,ab.
52 (weight loss maintenance adj (intervention$ or program$ or trial$)).ti,ab.
53 44 or 45 or 46 or 47 or 48 or 49 or 50 or 51 or 52
54 limit 53 to "all child (0 to 18 years)"
55 limit 53 to "all adult (19 plus years)"
56 54 not 55
57 53 not 56
58 limit 57 to animals
59 limit 57 to humans
60 58 not 59
61 57 not 60
62 clinical trials as topic/ or controlled clinical trials as topic/ or randomized controlled trials as topic/ or meta-analysis as topic/
63 (clinical trial or controlled clinical trial or meta analysis or randomized controlled trial).pt.
64 Random$.ti,ab.
65 control groups/ or double-blind method/ or single-blind method/
66 clinical trial$.ti,ab.
67 controlled trial$.ti,ab.
68 meta analy$.ti,ab.
69 62 or 63 or 64 or 65 or 66 or 67 or 68
70 61 and 69
71 limit 70 to english language
72 limit 71 to yr="2017 -Current"

For peer review only

PsycInfo

1 obesity
2 obese
3 overweight
4 weight loss
5 1 or 2 or 3 or 4
6 weight control/
7 behavior therapy/
8 cognitive behavior therapy/
9 cognitive therapy/
10 Cognitive Techniques/
11 Behavior Modification/
12 Behavior Change/
13 Motivational Interviewing/
14 counseling/
15 counselling.id.
16 Diets/
17 Dietary Restraint/
18 Exercise/
19 Physical Activity/
20 Aerobic Exercise/
21 Walking/
22 6 or 7 or 8 or 9 or 10 or 11 or 12 or 13 or 14 or 15 or 16 or 17 or 18 or 19 or 20 or 21
23 5 and 22
24 random$.ti,ab,id,hw.
25 placebo$.ti,ab,hw,id.
26 controlled trial$.ti,ab,id,hw.
27 clinical trial$.ti,ab,id,hw.
28 meta analy$.ti,ab,hw,id.
29 metaanaly$.ti,ab,hw,id.
30 24 or 25 or 26 or 27 or 28 or 29
31 23 and 30
32 limit 31 to ("300 adulthood <age 18 yrs and older>" or 320 young adulthood <age 18 to 29 yrs> or 340 thirties <age 30 to 39 yrs> or 360 middle age <age 40 to 64 yrs> or "380 aged <age 65 yrs and older>" or "390 very old <age 85 yrs and older>")
33 limit 68 to (english language and yr="2010 -Current")

For peer review only

BMJ Open

Inequalities in the uptake of, adherence to and effectiveness of behavioural weight management interventions: systematic review protocol

Journal:	*BMJ Open*
Manuscript ID	bmjopen-2020-039518.R1
Article Type:	Protocol
Date Submitted by the Author:	22-Oct-2020
Complete List of Authors:	Birch, Jack; University of Cambridge School of Clinical Medicine, MRC Epidemiology Unit<br>Griffin, Simon; University of Cambridge School of Clinical Medicine, MRC Epidemiology Unit; University of Cambridge School of Clinical Medicine, Primary Care Unit, Institute of Public Health<br>Kelly, Michael; University of Cambridge School of Clinical Medicine, Primary Care Unit<br>Ahern, Amy L.; University of Cambridge Department of Public Health and Primary Care, MRC Epidemiology
**Primary Subject Heading**:	Public health
Secondary Subject Heading:	Health services research
Keywords:	PRIMARY CARE, PUBLIC HEALTH, PREVENTIVE MEDICINE

SCHOLARONE™
Manuscripts

I, the Submitting Author has the right to grant and does grant on behalf of all authors of the Work (as defined in the below author licence), an exclusive licence and/or a non-exclusive licence for contributions from authors who are: i) UK Crown employees; ii) where BMJ has agreed a CC-BY licence shall apply, and/or iii) in accordance with the terms applicable for US Federal Government officers or employees acting as part of their official duties; on a worldwide, perpetual, irrevocable, royalty-free basis to BMJ Publishing Group Ltd ("BMJ") its licensees and where the relevant Journal is co-owned by BMJ to the co-owners of the Journal, to publish the Work in this journal and any other BMJ products and to exploit all rights, as set out in our licence.

The Submitting Author accepts and understands that any supply made under these terms is made by BMJ to the Submitting Author unless you are acting as an employee on behalf of your employer or a postgraduate student of an affiliated institution which is paying any applicable article publishing charge ("APC") for Open Access articles. Where the Submitting Author wishes to make the Work available on an Open Access basis (and intends to pay the relevant APC), the terms of reuse of such Open Access shall be governed by a Creative Commons licence – details of these licences and which Creative Commons licence will apply to this Work are set out in our licence referred to above.

Other than as permitted in any relevant BMJ Author's Self Archiving Policies, I confirm this Work has not been accepted for publication elsewhere, is not being considered for publication elsewhere and does not duplicate material already published. I confirm all authors consent to publication of this Work and authorise the granting of this licence.

For peer review only

Inequalities in the uptake of, adherence to and effectiveness of behavioural weight management interventions: systematic review protocol

Jack M. Birch[1], Simon J. Griffin[1, 2], Michael P Kelly[2], Amy L. Ahern[1]

Corresponding author

Jack M. Birch

MRC Epidemiology Unit, University of Cambridge School of Clinical Medicine, Box 285 Institute of Metabolic Science, Cambridge Biomedical Campus, CB2 0QQ

jack.birch@mrc-epid.cam.ac.uk

Author affiliations

[1] MRC Epidemiology Unit, University of Cambridge, Cambridge, UK.

[2] Primary Care Unit, Institute of Public Health, University of Cambridge, Cambridge, UK.

Full text word count 3767/4000 words

For peer review only - http://bmjopen.bmj.com/site/about/guidelines.xhtml

ABSTRACT

Introduction: It has been suggested that interventions focusing on individual behaviour change, such as behavioural weight management interventions, may exacerbate health inequalities. These intervention-generated inequalities may occur at different stages, including intervention uptake, adherence and effectiveness. We will synthesise evidence on how different measures of inequality moderate the uptake, adherence and effectiveness of behavioural weight management interventions in adults.

Methods and analysis: We will update a previous systematic literature review from the United States Preventive Services Taskforce to identify trials of behavioural weight management interventions in adults aged 18 and over that were, or could feasibly be, conducted in or recruited from primary care. Medline, Cochrane database (CENTRAL) and PsycINFO will be searched. Only RCTs and cluster-RCTs will be included. Two investigators will independently screen articles for eligibility and conduct risk of bias assessment. We will curate publication families for eligible trials. The PROGRESS-Plus acronym (place of residence, race/ethnicity, occupation, gender, religion, education, socioeconomic status, social capital, plus other discriminating factors) will be used to consider a comprehensive range of health inequalities. Data on trial uptake, intervention adherence, weight change, and PROGRESS-Plus related-data will be extracted. Data will be synthesised narratively. We will present a Harvest plot for each PROGRESS-Plus criterion and whether each trial found a negative, positive or no health inequality gradient. We will also identify potential sources of unpublished original research data on these factors which can be synthesised through a future individual participant data meta-analysis.

Ethics and dissemination: Ethical approval is not required as no primary data are being collected. The completed systematic review will be disseminated in a peer-reviewed journal, at conferences, and contribute to the lead author's PhD thesis. Authors of trials included in the completed systematic review may be invited to collaborate on a future individual participant data meta-analysis.

PROSPERO registration number: CRD42020173242

For peer review only

ARTICLE SUMMARY

Strengths and limitations of the review

- A description of existing data that relate to inequalities in behavioural weight management trials, and where they occur will be provided, enabling future meta-analysis of individual participant data.
- A comprehensive search strategy, which has previously been validated in the literature, will be used to identify relevant trials.
- Where data permit, subgroup analysis of association or interaction between the PROGRESS-Plus criteria and trial uptake, adherence and effectiveness will be presented in a Harvest plot.
- Only RCTs and cluster RCTs will be included
- There is likely heterogeneity in measures of PROGRESS-Plus criteria used, as well as limited publication of data, which is likely to prevent a meta-analysis being conducted.

For peer review only

For peer review only - http://bmjopen.bmj.com/site/about/guidelines.xhtml

INTRODUCTION

Rationale

Overweight and obesity are associated with an increased risk of a number of non-communicable diseases such as type 2 diabetes, cardiovascular disease and some cancers (including post-menopausal breast, bowel and oesophageal).[1, 2] People living with overweight and obesity have greater all-cause mortality compared to those within a healthy weight range.[3] There are known health inequalities by place of residence, ethnicity, occupation, sex, religion, education, socioeconomic status [SES], social capital and other factors such as disability and sexual orientation (PROGRESS-Plus).[4] Observational research suggests that inequalities in overweight and obesity exist across several of these criteria, such as SES and education,[5-10] although these measures are generally more predictive of obesity in women than men.[9, 11]

Both 'upstream' and 'downstream' interventions are needed to reduce the prevalence of obesity through primary prevention and treatment for those living with overweight and obesity. There is suggestion that 'upstream' interventions, i.e. those aimed at a population-level and requiring little personal agency, are the most equitable,[12] and may reduce inequalities in overweight and obesity prevalence. On the other hand, 'downstream' interventions, targeted at high-risk groups and individuals (such as those who already have overweight or obesity) and requiring high personal agency, are likely to be inequitable. Inequitable interventions may exacerbate health inequalities if they are less effective at reducing overweight and obesity prevalence in disadvantaged groups. Behavioural weight management interventions, such as those provided in or referred to from primary care, require a high level of personal agency as participants are required to attend and be engaged with an intervention for it to be effective.[13] Hence, behavioural weight management interventions may inadvertently exacerbate health inequalities.

The overall effectiveness of behavioural weight management interventions was considered in a systematic review and meta-analysis for the United States Preventive Services Task Force[USPSTF].[14] The review considered behavioural weight loss and behavioural weight loss maintenance interventions, as well as pharmacological weight loss and weight loss maintenance interventions. It found that primary care-relevant behavioural weight loss interventions were associated with greater mean weight loss at 12-18 months when compared to a control, whilst behavioural weight loss maintenance interventions are effective at preventing weight regain. Moderation of effectiveness by any of the PROGRESS-Plus criteria was not considered, although narrative comment was made about the reporting of ethnicity and SES. Unless a specific ethnicity was targeted in the intervention, the authors found that ethnicity and SES were not well reported. Where ethnicity and SES were reported, most participants were white and of mid-to-high SES. Income, employment and/or occupation were the most frequently reported measures of SES.

We identified one previous systematic review from Hillier-Brown *et al* that considered the effectiveness of individual, community and societal-level interventions at reducing socioeconomic inequalities in obesity.[11] The individual (n=5) and community interventions (n=12) included in the systematic review were similar to the behavioural interventions included in the USPSTF review. They defined individual-level interventions as being conducted in a healthcare, research or home setting and delivered one-to-one. Community-level interventions were defined as being delivered to a group and taking place in community settings such as in community or sports centres. The review found that, for individual-level interventions, evidence for reducing inequalities in obesity among adults was only found in tailored weight loss programmes targeted at low-income groups, particularly those in primary care

For peer review only - http://bmjopen.bmj.com/site/about/guidelines.xhtml

settings, rather than for 'universal' interventions. Evidence was generally only for short term outcomes (up to 9 months). Community-level interventions showed positive effects up to 3 months, although there was no evidence for longer term positive effects. Meanwhile, there was little evidence for the impact of societal-level ('upstream') interventions on inequalities in obesity among adults, and the included evidence was of low quality.

There are some limitations of the Hillier-Brown *et al* review. A meta-analysis was not conducted, due to heterogeneity of the studies. Whilst a highly sensitive search strategy was used, only literature that reported differential effects by a measure of SES were included. This meant that interventions which may have collected data on SES but had not included it in analyses reported in a published paper would have been excluded. The authors highlighted that this may have explain why mostly interventions that took a targeted approach to reducing SES inequalities were included; only a minority of studies examined intervention effects across the SES gradient. They also say that this targeted approach "has limitations as even when interventions are effective among low-income groups they are only able to reduce the health inequalities gap, they have little effect on the wider social gradient". Literature published since Hillier-Brown *et al* has considered the effect of universal interventions on health inequalities amongst adults.[15, 16]

The Hillier-Brown *et al* review only considered inequalities in intervention effectiveness. Intervention-generated inequalities may occur at several stages.[12] Firstly, in intervention uptake.[17] This may occur because of differing levels of weight loss service provision by geography or because some groups are less likely to take up the offer of an intervention. Research using the Clinical Practice Research Datalink in the United Kingdom [UK] found that certain groups, such as those in deprivation, may be more likely to access weight management interventions,[18] suggesting that such interventions may have a positive effect on health inequalities. Secondly, inequalities may occur in the adherence to an intervention.[19] Adherence to an intervention may be affected by certain barriers such as access to transport,[20] insufficient time or other social circumstances. Thirdly, there may be inequalities in outcome –those of a certain socioeconomic position or ethnicity may have similar uptake and adherence to an intervention, but there may be other factors that mean that the intervention is less effective for them than for other people. This may be because the intervention is not appropriately culturally or contextually tailored.

In the current systematic review, we will synthesise literature on inequalities across the uptake, adherence and effectiveness of behavioural weight management interventions The lack of reporting or analysis by measures associated with the PROGRESS-Plus criteria identified in both the USPSTF and Hillier Brown *et al's* systematic reviews suggest that it is not possible to fully explore inequalities in the effectiveness using aggregated data from published literature alone. This lack of reporting may have occurred because individual trials may not be sufficiently powered to detect an interaction between moderators such as SES and the outcome; they are likely just to be sufficiently powered to detect the main, overall effect. This systematic review will also identify trials with unpublished data on measures of inequality across the PROGRESS-Plus criteria in order to conduct a future individual participant data meta-analysis.

Objectives

The overall aim is to identify and describe inequalities in the update, adherence and effectiveness of behavioural weight management interventions. We will meet this aim through the following objectives: 1) to synthesise published literature on how inequalities across different PROGRESS-Plus criteria moderate the uptake, adherence and effectiveness of behavioural weight management interventions and;

For peer review only - http://bmjopen.bmj.com/site/about/guidelines.xhtml

2) to identify published trials that have unpublished data on how inequalities across different PROGRESS-Plus criteria moderate the uptake, adherence and effectiveness of behavioural weight management interventions.

METHODS

This protocol was written in accordance with the PRISMA-P reporting guidelines for systematic review protocols (supplementary file A) [21] and is registered on PROSPERO (CRD42020173242).

Study Design

We will conduct a systematic review of published randomised controlled trials [RCTs] of behavioural weight management interventions (which includes interventions for both behavioural weight loss and behavioural weight loss maintenance). PROGRESS-Plus criteria-related measures and data (outlined in Table 1) will be extracted and evidence regarding their impact on uptake, adherence and effectiveness will be synthesised. Furthermore, we will identify where data relating to the PROGRESS-Plus criteria have been collected and their relationship with uptake, adherence and effectiveness not analysed, to facilitate future individual participant data meta-analysis.

Initially, relevant literature concerning behavioural weight management trials will be extracted from the 2018 USPSTF systematic review of interventions to prevent obesity-related morbidity and mortality in adults.[14] Then, a search of the same databases used in the USPSTF review will be conducted to identify trials published since the search was completed for the USPSTF systematic review on 6th June 2017. We will use the same search strategies and terms as in the original report, but with pharmacological interventions excluded and terms relating to adverse events removed.

For peer review only

Table 1 Definition of PROGRESS-Plus factors (Adapted from Attwood et al)

PROGRESS-Plus Factor	Description	Example measures
**Place of residence**	Places, and perceptions of, where individuals live	• Postcode<br>• Country, state, region, town or community<br>• Urban/rural<br>• Housing characteristics<br>• Distance to attend weight loss session<br>• Local food environment<br>• 'Walkability'
**Race/ethnicity**	Racial or ethnic group, or other classification of culture, language or nationality status	• Ethnicity classifications<br>• Country of origin<br>• Language<br>• Other classifications of culture
**Occupation**	Occupational situation, patterns of work or features of working environment	• Professional/skilled/unskilled/unemployed<br>• Unemployed/employed/retired<br>• Full time/part time<br>• Manual/non-manual
**Gender/sex**	Gender is self-identified by individuals, incorporating ideas around socially constructed roles and behaviours<br><br>Sex refers to biological and physiological characteristics that define an individual as a man or woman	• Gender<br>• Sex (e.g. male/female classifications)
**Religion**	Religious affiliation or system of religious/spiritual beliefs or values	• Religious denomination
**Education**	Extent and type of education or other formal training	• Years in education<br>• Level of education attained (e.g. for UK: GCSE's, A-Levels, Undergraduate)<br>• Institutions attended (e.g. for USA: high school/some college/college graduate/university)
**Socioeconomic status**	An individual's position within a hierarchical social structure. Measures of socioeconomic status aim to capture access to resources, privilege, power or control	• Indices of Multiple Deprivation (IMD, UK only, Scottish Index of Multiple Deprivation)<br>• Social class<br>• Individual income<br>• Household income<br>• Receiving state welfare (e.g. benefits/free prescriptions in the UK, Medicaid in the USA)

For peer review only

		• Asset-based measures (e.g. home or car ownership)<br>• Occupation (e.g. occupation class)
**Social capital**	Social capital aims to capture the obligations and benefits conferred upon an individual by their society and social relationships. Can be viewed as a measure of interconnectedness between an individual and their social surroundings or group	• Marital/relationship status (e.g. single, cohabiting)<br>• Household size<br>• Social support<br>• Social networks<br>• Civic participation/group membership<br>• Ability to use technology
**Plus**	Any other factors over an individual's life course that could lead to discrimination. Examples include age, disability and sexual orientation	• Self-reported age in years<br>• Measures of health status and/or quality of life (e.g. EuroQoL, SF-36, EQ5D)<br>• Tests of physical function<br>• Physical or emotional/mental disability<br>• Self-reported sexual orientation (e.g. heterosexual, homosexual, bisexual)

Eligibility criteria

We will select studies according to the criteria outlined below.

Study designs

We will include research articles reporting randomised controlled trials (RCTs) and cluster-RCTs. To mirror the USPSTF review, only studies published in the English language will be included.

Participants

We will include studies of adults aged 18 years and over with overweight or obesity (BMI $\geq$ 25 kg/m^2) who are suitable for behavioural weight loss or behavioural weight loss maintenance interventions. Participants may have additional risk factors such as hypertension, dyslipidaemia, impaired glucose tolerance or impaired fasting glucose.

Studies will be excluded if the population: was not selected based on a weight-related measure; had secondary causes of obesity (such as steroid use); selected on the basis of having a chronic disease for which behavioural weight loss or behavioural weight loss maintenance is part of disease management; was of pregnant women; was of adults in institutions; or if the intervention was targeted at parents in order to change the behaviour of children.

Interventions

Studies will be included if they were conducted in or recruited from primary care or a healthcare system, or could feasibly be implemented in or referred to from primary care. In the case of the latter, the interventions must be conducted as part of a healthcare setting or be available in the community at a national level, such as commercial weight loss interventions. We will include behavioural interventions that are focused on weight loss or weight loss maintenance. Interventions may be delivered either alone

For peer review only - http://bmjopen.bmj.com/site/about/guidelines.xhtml

or as part of a multicomponent intervention on wider diet and nutrition, physical activity, sedentary behaviour or a combination of these. The intervention may include (but not limited to): assessment with feedback, advice, collaborative goal-setting, assistance, exercise prescriptions (referral to exercise facility or programme), arranging further contacts or provider training.

The delivery of the intervention may be: face-to-face contact, telephone, print materials, or be computer or mobile-phone based technology (such as websites, apps or text messages). There is no restriction on who delivers the intervention.

Interventions of alternative and complementary treatments (e.g. mindfulness) will be excluded. All pharmacological and surgical interventions will be excluded, including in combination with behavioural interventions, unless the trial includes behavioural only and control arms.

Comparators

We will only include trials with a control group. The control group may receive no intervention (wait-list control or usual care) or minimal intervention (such as generic print or electronic materials).

Outcomes and prioritisation

Outcomes will occur at the three stages: uptake, attendance/adherence, and effectiveness.

Differential uptake will be considered at two stages. Firstly, trial uptake will be calculated for each study using the formula:

$$\frac{Participants\ accepting\ invitation\ to\ trial}{Participants\ invited\ to\ trial}$$

Secondly, uptake of the intervention arm. We are considering uptake of the intervention to be 'attending' at least one intervention session, the language of which is geared towards to those attending community group interventions such as WW (formerly Weight Watchers). 'Attendance' to an online-based intervention would be defined as logging into the online platform at least once. Hence, uptake of the intervention is defined as:

$$\frac{Participants\ attending\ at\ least\ one\ intervention\ session}{Participants\ in\ trial\ arm}$$

The second outcome stage is adherence to the intervention. We will consider adherence for each participant as either a binary variable (adhered versus not adhered) or using the below formula, depending on how adherence is defined in the included studies.

$$\frac{Number\ of\ sessions\ attended}{Number\ of\ sessions\ prescribed}$$

The final outcome stage is effectiveness. This will be assessed at the 12-month follow up using three measures: weight change in kilograms, weight loss of five percent or greater, and change in waist circumference.

Timing (e.g. minimum follow up)

As per the USPSTF review, we will only include studies that measure intervention effectiveness at 12 or 18 months. This is despite the different timing required for each of the outcomes. The uptake

For peer review only - http://bmjopen.bmj.com/site/about/guidelines.xhtml
For peer review only

outcomes require data at two pre-intervention stages – invitation to trial and baseline – as well as data on percentage of participants attending at least one session of an intervention. The adherence outcome will require data from baseline until the end of the intervention. Finally, intervention effectiveness will be assessed at 12 months or later from baseline.

Moderator variables

The moderator variables under consideration are the PROGRESS-Plus criteria. Possible measures of each of these criteria are shown in Table 1, which has been adapted from a systematic review that explored equity in primary care-based physical activity interventions using PROGRESS-Plus. [22]

Setting

Eligible studies will have been conducted in primary care, referred from primary care, or be applicable to primary care settings. As per the search performed in the USPSTF review, only studies conducted in countries that were members of the Organisation for Economic Co-operation and Development (as of 2017) are eligible for inclusion. These countries are: Australia, Austria, Belgium, Canada, Chile, Czech Republic, Denmark, Estonia, Finland, France, Germany, Greece, Hungary, Iceland, Israel, Italy, Japan, South Korea, Luxembourg, Mexico, the Netherlands, New Zealand, Norway, Poland, Portugal, Slovakia, Slovenia, Spain, Sweden, Switzerland, Turkey, United Kingdom and the United States.

Information sources and search strategy

Electronic searches

It is anticipated that much of the PROGRESS-Plus data to be extracted will not be reported in the main write up of each behavioural weight management intervention RCT. Hence, it is necessary to extract data from all publications associated with each individual RCT. To complete this, we will adopt a similar approach to literature searching as demonstrated by Orkin *et al*.[23]

The search strategy will be completed in two phases. Phase 1 is identifying 'parent' RCTs. These studies will be identified in two ways. Initially, the behavioural weight loss and behavioural weight loss maintenance interventions included in the USPSTF report will be extracted. Then, we will conduct an update of the literature search used in the USPSTF report to capture recent published trials using the same databases (Medline, CENTRAL and PsychInfo). Databases will be searched from June 2017 (the last date of the USPSTF report) to February 2020. The search strategy includes the following concepts: 1) overweight and obesity AND 2) behavioural weight loss/ behavioural weight loss maintenance interventions. The Medline, CENTRAL and PsychInfo search strategies are outlined in supplementary file B. In addition to the databases, reference lists of included published primary research and relevant systematic reviews and meta-analyses will be searched for possible further studies for inclusion.

Phase 2 is to "curate publication families".[23] This involves identifying publications of any type that relate to the parent RCT through electronic database searching. Authors and study identifiers (such as trial name) will be extracted from each parent RCT, which will then be searched for in the same electronic bibliographic databases as phase 1. Each publication family will be considered as one study.

Study records

Data management and study selection

For peer review only - http://bmjopen.bmj.com/site/about/guidelines.xhtml

Search results will be imported into Endnote X7 bibliographic software, where duplicates will be removed. The literature will then be loaded onto Covidence systematic review software (Veritas Health Innovation, Melbourne, Australia), and title and abstract screening conducted. Piloting of 500 articles will be conducted with minimum two investigators, where differences in interpretation of the inclusion criteria will be discussed between the investigators in order to achieve consistency in the review process. Once this has been completed, the remaining titles and abstracts will be screened for inclusion by minimum two investigators independently. Full-text articles identified as being potentially relevant to the research questions will be accessed and screened by minimum two investigators. Any conflicts will be discussed and resolved by a third reviewer if agreement cannot be reached. For articles excluded at full-text stage, reasons for exclusion will be recorded.

Multiple articles reporting the same study will all be included and amalgamated to ensure all the best available data are used. A PRISMA flow chart will be reported to visualise the study selection.[24]

Data items

For studies highlighted as eligible for inclusion from the USPSTF report, and those that fulfil the inclusion criteria from our subsequent searches, we will extract data from the reports onto a data extraction form. To ensure that an appropriate breadth and depth of detail is captured, the data extraction form will be based on the Cochrane Public Health Group data extraction form,[25] the Consolidated Standards of Reporting Trials 2010 statement,[26] the Template for Intervention Description and Replication checklist and the PROGRESS-Plus criteria.[4, 27]

The following data will be extracted from the studies:

- General information (study authors, publication year, country and source of funding)
- Study information (study aim, design, recruitment location and method, randomisation, blinding and allocation concealment)
- Participant information (measures associated with the PROGRESS-Plus criteria as outlined in Table 1)
- Intervention information (content, delivery method, group or individual-level, duration, setting, profession of person delivering intervention)
- Comparator information (control/usual care, content, delivery method, group or individual-level, duration, setting, profession of person delivering intervention)
- Uptake (number of participants invited to trial, number of participants accepting invite, number of participants randomised to intervention arm, number of participants attending $\geq$ 1 session), including impact of PROGRESS-Plus criteria on uptake
- Attrition/adherence/attendance, including impact of PROGRESS-Plus criteria on these
- Outcomes (outcomes studies, self-report or objective, follow-up duration, statistical analyses, intervention effect sizes), including impact of PROGRESS-Plus criteria on outcomes

We will contact authors where data relating to the uptake, adherence and effectiveness outcomes have not been published. The corresponding author for each study will be contacted by email, and followed up after two weeks if no response is received. One month from the initial email will be allowed for study authors to respond.

Risk of bias in individual studies

We will use Cochrane's risk of bias tool for randomised trials (RoB 2) to assess risk of bias across all included studies.[28] This ensures all included studies are assessed by the same criteria for the risk of

For peer review only - http://bmjopen.bmj.com/site/about/guidelines.xhtml

bias. The tool covers six domains of possible bias: the randomisation process; allocation concealment; participant and trial personnel blinding; blinding of outcome assessment; incomplete outcome data; and selective reporting. Each domain is given a ranking of 'low risk', 'high risk', or 'unclear. This will be performed independently by at least two study authors. Where disagreements occur, these will be discussed between authors to reach consensus. A third reviewer will be consulted if agreement cannot be reached. Other possible sources of bias that does not fall within RoB 2's six domains will be noted by reviewers, and commented on if appropriate in the final review. Reviewers will not be blinded to study information (such as study author, institution or journal). Results of the risk of bias assessments will be presented in a summary figure outlining a study's overall risk of bias as well as the risk of bias in each domain.

Data synthesis

Narrative synthesis and Harvest plots

We anticipate that there will be insufficient data to conduct a meta-analysis, therefore, the primary methods of data synthesis will be narrative analysis and using Harvest plots.[29] Harvest plots were proposed by Ogilvie *et al* as a method for synthesising evidence of the differential effectiveness of population-level public interventions,[29] but have been used in systematic reviews of various intervention types since.[30-35] Even where there is heterogeneity in measures used, Harvest plots allow for all available and relevant data to be used and presented.[29, 36, 37] Several study features can be graphically demonstrated on a single plot, such as study quality, statistical significance and sample size. We will present a Harvest plot for each PROGRESS-Plus criteria and whether each trial found a negative, positive or no health inequality gradient; sample size of each study group; and whether the trial considered an intervention or interaction effect on the health inequality gradient.

Meta-analysis

Should there be sufficient data to conduct a meta-analysis, then the meta-analysis will consider two questions: *are the PROGRESS-Plus criteria associated with the amount of weight loss achieved following behavioural weight management intervention?* and *do the PROGRESS-Plus criteria moderate the effectiveness of behavioural weight management interventions?*. Odds or risk ratios would be pooled for each question; the first question assesses if there is an association between the PROGRESS-Plus criteria and weight loss, the second question considers if there is an interaction. The data would be analysed using Stata v16 (StataCorp. 2019. College Station, TX: StataCorp LLC), using a random-effects meta-analysis.

Statistical heterogeneity will be assessed using the I^2 statistic and its 95% confidence interval. The I^2 statistic will be interpreted against the following categorisations: 0% to 40% might not be important; 30% to 60% may represent moderate heterogeneity; 50% to 90% may represent substantial heterogeneity and; 75% to 100% is likely considerable heterogeneity.[38] The overlap in these categories exist as they are not intended as absolute threshold judgements, but as a guide to be used in conjunction with possible reasons explaining variability [38]. Publication bias will be considered using a funnel plot.

Patient and public involvement

A patient and public involvement representative reviewed a lay summary of our proposed plan for the systematic review. Feedback was received on the review's aims and definitions of the PROGRESS-Plus criteria. Once the review has been completed, feedback will be sought from the patient and public

For peer review only - http://bmjopen.bmj.com/site/about/guidelines.xhtml

involvement representatives about the interpretation of findings and plans for an individual participant data meta-analysis.

ETHICS AND DISSEMINATION

Ethical approval is not required as only aggregate data are going to be acquired, and will be used for the purpose for which they were originally collected for. Ethical approval for each trial to be included will have been sought by the original investigators. This systematic review and meta-analysis will follow the PRISMA statement.[24]

Inequalities in overweight and obesity, and in health promotion interventions, are widely recognised. However, inequalities in behavioural weight loss interventions delivered or referred to from primary care (or similar) have not yet been considered in a systematic review. This review will identify data on where inequalities in weight loss interventions occur (i.e. in which PROGRESS-Plus criteria), and at what stage (uptake, adherence or effectiveness). We anticipate the completed systematic review will be published in a scientific journal, presented at conferences and contribute to the lead author's PhD thesis. The review findings will contribute towards the consideration of intervention-generated inequalities by researchers, policy makers and healthcare and public health practitioners.

Acknowledgements

The authors would like to thank Hazel Patel, the patient and public involvement representative, who commented on the lay summary of our proposed plan, for their contribution in the development of this research.

Competing interests

ALA is principal investigator on two publicly funded (NIHR, MRC) trials where the intervention is provided by WW (formerly Weight Watchers) at no cost. MPK has undertaken consultancy for Slimming World, and led the clinical and public health guidelines development for NICE from 2005 until 2014.

Author contributions

JMB conceived and designed the study, developed the search strategy and drafted the manuscript. SJG conceived the study, contributed to study design and reviewed drafts of the manuscript. MPK contributed to study design and reviewed drafts of the manuscript. ALA conceived the study, contributed to study design and reviewed drafts of the manuscript. All authors have reviewed the manuscript and approved the final version for publication.

Funding

JMB, SJG and ALA are supported by the Medical Research Council (MRC) (Grant MC_UU_12015/4). The University of Cambridge has received salary support in respect of SJG from the National Health Service in the East of England through the Clinical Academic Reserve.

For peer review only - http://bmjopen.bmj.com/site/about/guidelines.xhtml

REFERENCES

1. Lauby-Secretan B, Scoccianti C, Loomis D, et al. Body Fatness and Cancer — Viewpoint of the IARC Working Group. New England Journal of Medicine. 2016;375(8):794-8.
2. Forouzanfar MH, Afshin A, Alexander LT, et al. Global, regional, and national comparative risk assessment of 79 behavioural, environmental and occupational, and metabolic risks or clusters of risks, 1990–2015: a systematic analysis for the Global Burden of Disease Study 2015. The Lancet. 2016;388(10053):1659-724.
3. Di Angelantonio E, Bhupathiraju SN, Wormser D, et al. Body-mass index and all-cause mortality: individual-participant-data meta-analysis of 239 prospective studies in four continents. The Lancet. 2016;388(10046):776-86.
4. O'Neill J, Tabish H, Welch V, et al. Applying an equity lens to interventions: using PROGRESS ensures consideration of socially stratifying factors to illuminate inequities in health. Journal of clinical epidemiology. 2014;67(1):56-64.
5. Newton S, Braithwaite D, Akinyemiju TF. Socio-economic status over the life course and obesity: Systematic review and meta-analysis. PLoS One. 2017;12(5):e0177151-e.
6. Loring B, Robertson A. Guidance for addressing inequities in overweight and obesity. Copenhagen: Word Health Organization Regional Office for Europe; 2014.
7. Vilhelmsson A, Östergren P-O. Reducing health inequalities with interventions targeting behavioral factors among individuals with low levels of education - A rapid review. PLoS One. 2018;13(4):e0195774-e.
8. Clarke P, O'Malley PM, Johnston LD, et al. Social disparities in BMI trajectories across adulthood by gender, race/ethnicity and lifetime socio-economic position: 1986-2004. Int J Epidemiol. 2009;38(2):499-509.
9. El-Sayed AM, Scarborough P, Galea S. Unevenly distributed: a systematic review of the health literature about socioeconomic inequalities in adult obesity in the United Kingdom. BMC public health. 2012;12(1):18.
10. Rohrmann S, Steinbrecher A, Linseisen J, et al. The association of education with long-term weight change in the EPIC-PANACEA cohort. European Journal of Clinical Nutrition. 2012;66(8):957-63.
11. Hillier-Brown FC, Bambra CL, Cairns JM, et al. A systematic review of the effectiveness of individual, community and societal-level interventions at reducing socio-economic inequalities in obesity among adults. International journal of obesity (2005). 2014;38(12):1483-90.
12. White M, Adams J, Heywood P. How and why do interventions that increase health overall widen inequalities within populations? . In: Babones SJ, editor. Social inequality and public health. Bristol: Policy Press; 2009.
13. Adams J, Mytton O, White M, et al. Why Are Some Population Interventions for Diet and Obesity More Equitable and Effective Than Others? The Role of Individual Agency. PLOS Medicine. 2016;13(4):e1001990.
14. LeBlanc ES, Patnode CD, Webber EM, et al. Behavioral and Pharmacotherapy Weight Loss Interventions to Prevent Obesity-Related Morbidity and Mortality in Adults: Updated Evidence Report and Systematic Review for the US Preventive Services Task Force. Jama. 2018;320(11):1172-91.
15. Graham J, Tudor K, Jebb SA, et al. The equity impact of brief opportunistic interventions to promote weight loss in primary care: secondary analysis of the BWeL randomised trial. BMC Med. 2019;17(1):51-.
16. Greaves C, Gillison F, Stathi A, et al. Waste the waist: a pilot randomised controlled trial of a primary care based intervention to support lifestyle change in people with high cardiovascular risk. International Journal of Behavioral Nutrition and Physical Activity. 2015;12(1):1.
17. Ahern AL, Aveyard P, Boyland EJ, et al. Inequalities in the uptake of weight management interventions in a pragmatic trial: an observational study in primary care. British Journal of General Practice. 2016;66(645):e258-e63.
18. Booth HP, Prevost AT, Gulliford MC. Access to weight reduction interventions for overweight and obese patients in UK primary care: population-based cohort study. BMJ Open. 2015;5(1):e006642.

For peer review only - http://bmjopen.bmj.com/site/about/guidelines.xhtml

19. Kure-Biegel N, Schnohr CW, Hindhede AL, et al. Risk factors for not completing health interventions and the potential impact on health inequalities between educational groups – a mixed method study from Denmark. International Journal for Equity in Health. 2016;15(1):54.
20. Austin JL, Smith JE, Gianini L, et al. Attitudinal familism predicts weight management adherence in Mexican–American women. J Behav Med. 2013;36(3):259-69.
21. Moher D, Shamseer L, Clarke M, et al. Preferred reporting items for systematic review and meta-analysis protocols (PRISMA-P) 2015 statement. Systematic Reviews. 2015;4(1):1.
22. Attwood S, van Sluijs E, Sutton S. Exploring equity in primary-care-based physical activity interventions using PROGRESS-Plus: a systematic review and evidence synthesis. Int J Behav Nutr Phys Act. 2016;13:60-.
23. Orkin AM, McArthur A, McDonald A, et al. Defining and measuring health equity effects in research on task shifting interventions in high-income countries: a systematic review protocol. BMJ Open. 2018;8(7):e021172.
24. Moher D, Liberati A, Tetzlaff J, et al. Preferred reporting items for systematic reviews and meta-analyses: the PRISMA statement. BMJ. 2009;339.
25. The Cochrane Public Health Group. Cochrane public health group data extraction and assessment template. 2011.
26. Schulz KF, Altman DG, Moher D, et al. CONSORT 2010 Statement: updated guidelines for reporting parallel group randomised trials. BMC Med. 2010;8:18-.
27. Hoffmann TC, Glasziou PP, Boutron I, et al. Better reporting of interventions: template for intervention description and replication (TIDieR) checklist and guide. BMJ. 2014;348:g1687-g.
28. Sterne JAC, Savović J, Page MJ, et al. RoB 2: a revised tool for assessing risk of bias in randomised trials. BMJ. 2019;366:l4898.
29. Ogilvie D, Fayter D, Petticrew M, et al. The harvest plot: A method for synthesising evidence about the differential effects of interventions. BMC Medical Research Methodology. 2008;8(1):8.
30. Romppanen J, Häggman-Laitila A. Interventions for nurses' well-being at work: a quantitative systematic review. Journal of Advanced Nursing. 2017;73(7):1555-69.
31. Attwood S, van Sluijs E, Sutton S. Exploring equity in primary-care-based physical activity interventions using PROGRESS-Plus: a systematic review and evidence synthesis. The international journal of behavioral nutrition and physical activity. 2016;13:60.
32. Moore GF, Littlecott HJ, Turley R, et al. Socioeconomic gradients in the effects of universal school-based health behaviour interventions: a systematic review of intervention studies. BMC Public Health. 2015;15(1):907.
33. Wijndaele K, Lakshman R, Landsbaugh JR, et al. Determinants of Early Weaning and Use of Unmodified Cow's Milk in Infants: A Systematic Review. Journal of the American Dietetic Association. 2009;109(12):2017-28.
34. Kovacs E, Strobl R, Phillips A, et al. Systematic Review and Meta-analysis of the Effectiveness of Implementation Strategies for Non-communicable Disease Guidelines in Primary Health Care. J Gen Intern Med. 2018;33(7):1142-54.
35. Nanninga S, Lehne G, Ratz T, et al. Impact of Public Smoking Bans on Social Inequalities in Children's Exposure to Tobacco Smoke at Home: An Equity-Focused Systematic Review. Nicotine & Tobacco Research. 2018;21(11):1462-72.
36. Burns J, Polus S, Brereton L, et al. Looking beyond the forest: Using harvest plots, gap analysis, and expert consultations to assess effectiveness, engage stakeholders, and inform policy. Research Synthesis Methods. 2018;9(1):132-40.
37. Crowther M, Avenell A, MacLennan G, et al. A further use for the Harvest plot: a novel method for the presentation of data synthesis. Research synthesis methods. 2011;2(2):79-83.
38. Ryan R. Cochrane Consumers and Communication Review Group. 'Heterogeneity and subgroup analyses in Cochrane Consumers and Communication Group reviews: planning the analysis at protocol stage. Cochrane Consumers and Communication; 2016. Contract No.: 06/02/2020.

For peer review only - http://bmjopen.bmj.com/site/about/guidelines.xhtml

PRISMA-P (Preferred Reporting Items for Systematic review and Meta-Analysis Protocols) 2015 checklist: recommended items to address in a systematic review protocol*

Section and topic	Item No	Checklist item	Location in text
**ADMINISTRATIVE INFORMATION**			
Title:			
Identification	1a	Identify the report as a protocol of a systematic review	Page 1
Update	1b	If the protocol is for an update of a previous systematic review, identify as such	N/A
Registration	2	If registered, provide the name of the registry (such as PROSPERO) and registration number	Page 2 (abstract)
Authors:			
Contact	3a	Provide name, institutional affiliation, e-mail address of all protocol authors; provide physical mailing address of corresponding author	Page 1
Contributions	3b	Describe contributions of protocol authors and identify the guarantor of the review	Page 13
Amendments	4	If the protocol represents an amendment of a previously completed or published protocol, identify as such and list changes; otherwise, state plan for documenting important protocol amendments	N/A
Support:			
Sources	5a	Indicate sources of financial or other support for the review	Page 13
Sponsor	5b	Provide name for the review funder and/or sponsor	Page 13
Role of sponsor or funder	5c	Describe roles of funder(s), sponsor(s), and/or institution(s), if any, in developing the protocol	Page 13
**INTRODUCTION**			
Rationale	6	Describe the rationale for the review in the context of what is already known	Page 4-5
Objectives	7	Provide an explicit statement of the question(s) the review will address with reference to participants, interventions, comparators, and outcomes (PICO)	Page 5
**METHODS**			
Eligibility criteria	8	Specify the study characteristics (such as PICO, study design, setting, time frame) and report characteristics (such as years considered, language, publication status) to be used as criteria for eligibility for the review	Page 5-10
Information sources	9	Describe all intended information sources (such as electronic databases, contact with study authors, trial registers or other grey literature sources) with planned dates of coverage	Page 10
Search strategy	10	Present draft of search strategy to be used for at least one electronic database, including planned limits, such that it could be repeated	Supplementary file B

For peer review only

Study records:			
Data management	11a	Describe the mechanism(s) that will be used to manage records and data throughout the review	Page 10-11
Selection process	11b	State the process that will be used for selecting studies (such as two independent reviewers) through each phase of the review (that is, screening, eligibility and inclusion in meta-analysis)	Page 11
Data collection process	11c	Describe planned method of extracting data from reports (such as piloting forms, done independently, in duplicate), any processes for obtaining and confirming data from investigators	Page 11
Data items	12	List and define all variables for which data will be sought (such as PICO items, funding sources), any pre-planned data assumptions and simplifications	Page 11
Outcomes and prioritization	13	List and define all outcomes for which data will be sought, including prioritization of main and additional outcomes, with rationale	Page 9-10
Risk of bias in individual studies	14	Describe anticipated methods for assessing risk of bias of individual studies, including whether this will be done at the outcome or study level, or both; state how this information will be used in data synthesis	Page 11-12
Data synthesis	15a	Describe criteria under which study data will be quantitatively synthesised	Page 12
	15b	If data are appropriate for quantitative synthesis, describe planned summary measures, methods of handling data and methods of combining data from studies, including any planned exploration of consistency (such as I^2, Kendall's τ)	Page 12
	15c	Describe any proposed additional analyses (such as sensitivity or subgroup analyses, meta-regression)	Page 12
	15d	If quantitative synthesis is not appropriate, describe the type of summary planned	Page 12
Meta-bias(es)	16	Specify any planned assessment of meta-bias(es) (such as publication bias across studies, selective reporting within studies)	Page 12
Confidence in cumulative evidence	17	Describe how the strength of the body of evidence will be assessed (such as GRADE)	

*** It is strongly recommended that this checklist be read in conjunction with the PRISMA-P Explanation and Elaboration (cite when available) for important clarification on the items. Amendments to a review protocol should be tracked and dated. The copyright for PRISMA-P (including checklist) is held by the PRISMA-P Group and is distributed under a Creative Commons Attribution Licence 4.0.**

From: Shamseer L, Moher D, Clarke M, Ghersi D, Liberati A, Petticrew M, Shekelle P, Stewart L, PRISMA-P Group. Preferred reporting items for systematic review and meta-analysis protocols (PRISMA-P) 2015: elaboration and explanation. BMJ. 2015 Jan 2;349(jan02 1):g7647.

For peer review only

Search Strategies

Cochrane Central Register of Controlled Trials (CENTRAL)

#1 (weight or adipos*):ti or (obesity or obese or overweight or "weight loss"):ti,ab,kw
#2 behavio*:ti,ab,kw
#3 counsel*.ti,ab,kw
#4 cognitive:ti,ab,kw
#5 (diet* or nutrition*):ti,ab,kw
#6 (weightwatcher* or (weight next watcher*)):ti,ab,kw
#7 "physical activity":ti,ab,kw
#8 exercise:ti,ab,kw
#9 (lifestyle or "life style"):ti,ab,kw next (modification* or intervention*):ti,ab,kw
#10 (or #2-#9)
#11 #1 and #10
#12 "weight loss":ti,ab,kw next (intervention* or program* or trial*):ti,ab,kw
#13 (weight next reduc*):ti,ab,kw next (intervention* or program* or trial*):ti,ab,kw
#14 "weight management":ti,ab,kw next (intervention* or program* or trial*):ti,ab,kw
#15 "weight control":ti,ab,kw next (intervention* or program* or trial*):ti,ab,kw
#16 ("weight loss maintenance" next (intervention* or program* or trial*)):ti,ab,kw
#17 (or #11-#16)
#18 (child* or adolescen* or pediatric* or paediatric*)
#19 adult*
#20 (#18 not #19)
#21 (#17 not #20) Publication Year from 2017 to 2020, in Trials

For peer review only

Ovid Medline [ALL KQ]
1 Obesity/
2 Obesity, Morbid/
3 Overweight/
4 Obesity, Metabolically Benign/
5 Weight loss/
6 obes$.ti.
7 overweight.ti.
8 weight.ti.
9 (adipos$ or body fat).ti.
10 (obes$ or overweight or weight loss).ti,ab.
11 limit 10 to ("in data review" or in process or "pubmed not medline")
12 1 or 2 or 3 or 4 or 5 or 6 or 7 or 8 or 9 or 11
13 Weight Reduction Programs/
14 Behavior Therapy/
15 Cognitive Therapy/
16 Counseling/
17 Directive Counseling/
18 Self-Help Groups/
19 counsel$.ti,ab.
20 (behav$ adj3 (therap$ or program$ or intervention$)).ti,ab.
21 Health Education/
22 Diet, Reducing/
23 Diet, Fat-Restricted/
24 Caloric Restriction/
25 Diet Therapy/
26 (diet$ adj counsel$).ti,ab.
27 (diet$ adj education$).ti,ab.
28 (nutrition$ adj counsel$).ti,ab.
29 (nutrition$ adj education$).ti,ab.
30 (nutrition$ adj intervention$).ti,ab.
31 (diet$ adj (modif$ or therapy or intervention$ or strateg$)).ti,ab.
32 ((diet or dieting or slim$) adj (club$ or organi?ation$)).ti,ab.
33 (weight reduc$ adj diet$).ti,ab.
34 (weightwatcher$ or weight watcher$).ti,ab.
35 Exercise/
36 Exercise Therapy/
37 Motor Activity/
38 Physical Conditioning, Human/
39 Physical Fitness/
40 physical activity.ti,ab.
41 (exercise adj3 (therap$ or program$ or intervention$)).ti,ab.
42 ((lifestyle or life style) adj (modification$ or intervention$)).ti,ab.
43 13 or 14 or 15 or 16 or 17 or 18 or 19 or 20 or 21 or 22 or 23 or 24 or 25 or 26 or 27 or 28 or 29 or 30 or 31 or 32 or 33 or 34 or 35 or 36 or 37 or 38 or 39 or 40 or 41 or 42
44 12 and 43
45 Obesity/dh, th, dt, rh [Diet Therapy, Therapy, Drug Therapy, Rehabilitation]
46 Obesity, Morbid/dh, th, dt, rh
47 Overweight/dh, th, dt, rh
48 (weight loss adj (intervention$ or program$ or trial$)).ti,ab.
49 (weight reduc$ adj (intervention$ or program$ or trial$)).ti,ab.
50 (weight management adj (intervention$ or program$ or trial$)).ti,ab.

For peer review only

51 (weight control adj (intervention$ or program$ or trial$)).ti,ab.
52 (weight loss maintenance adj (intervention$ or program$ or trial$)).ti,ab.
53 44 or 45 or 46 or 47 or 48 or 49 or 50 or 51 or 52
54 limit 53 to "all child (0 to 18 years)"
55 limit 53 to "all adult (19 plus years)"
56 54 not 55
57 53 not 56
58 limit 57 to animals
59 limit 57 to humans
60 58 not 59
61 57 not 60
62 clinical trials as topic/ or controlled clinical trials as topic/ or randomized controlled trials as topic/ or meta-analysis as topic/
63 (clinical trial or controlled clinical trial or meta analysis or randomized controlled trial).pt.
64 Random$.ti,ab.
65 control groups/ or double-blind method/ or single-blind method/
66 clinical trial$.ti,ab.
67 controlled trial$.ti,ab.
68 meta analy$.ti,ab.
69 62 or 63 or 64 or 65 or 66 or 67 or 68
70 61 and 69
71 limit 70 to english language
72 limit 71 to yr="2017 -Current"

For peer review only

PsycInfo
1 obesity
2 obese
3 overweight
4 weight loss
5 1 or 2 or 3 or 4
6 weight control/
7 behavior therapy/
8 cognitive behavior therapy/
9 cognitive therapy/
10 Cognitive Techniques/
11 Behavior Modification/
12 Behavior Change/
13 Motivational Interviewing/
14 counseling/
15 counselling.id.
16 Diets/
17 Dietary Restraint/
18 Exercise/
19 Physical Activity/
20 Aerobic Exercise/
21 Walking/
22 6 or 7 or 8 or 9 or 10 or 11 or 12 or 13 or 14 or 15 or 16 or 17 or 18 or 19 or 20 or 21
23 5 and 22
24 random$.ti,ab,id,hw.
25 placebo$.ti,ab,hw,id.
26 controlled trial$.ti,ab,id,hw.
27 clinical trial$.ti,ab,id,hw.
28 meta analy$.ti,ab,hw,id.
29 metaanaly$.ti,ab,hw,id.
30 24 or 25 or 26 or 27 or 28 or 29
31 23 and 30
32 limit 31 to ("300 adulthood <age 18 yrs and older>" or 320 young adulthood <age 18 to 29 yrs> or 340 thirties <age 30 to 39 yrs> or 360 middle age <age 40 to 64 yrs> or "380 aged <age 65 yrs and older>" or "390 very old <age 85 yrs and older>")
33 limit 68 to (english language and yr="2010 -Current")

For peer review only
